# MR radiomics in assessment of consistency of pituitary macroadenoma: can T1-weighted contrast enhanced image improve diagnostic performance of T2-weighted image?

**DOI:** 10.3389/fonc.2025.1539432

**Published:** 2025-09-03

**Authors:** Menghong Zou, Hongwei Li, Hongchao Yao, Yang Liu, Jie Zhang

**Affiliations:** ^1^ Department of Radiology, The Third Hospital of Mianyang (Sichuan Mental Health Center), Mianyang, China; ^2^ Department of Neurosurgery, The Third Hospital of Mianyang (Sichuan Mental Health Center), Mianyang, China

**Keywords:** radiomics, pituitary macroadenoma, hierarchical analysis, consistency, combined model

## Abstract

**Objectives:**

To evaluate and compare the efficacy of radiomics models derived from T2-weighted and/or contrast-enhanced T1-weighted (CET1) images in assessing pituitary macroadenoma consistency, and to validate their performance stability under varying MRI field strengths and scanner vendors.

**Methods:**

A total of 133 patients with pathologically proven pituitary macroadenomas (35 fibrous, 98 non-fibrous) were retrospectively included. Three logistic regression models were constructed: a *T2 model*, a *CET1 model*, and a *T2-CET1 combined model*, based on features selected from coronal T2-weighted and contrast-enhanced T1-weighted (CET1) images. An external validation cohort of 40 patients (20 fibrous, 20 non-fibrous) was selected from another healthcare institution. Model performance was primarily evaluated using receiver operating characteristic (ROC) analysis. Stratified analyses were performed to compare the predictive performance of the models across different magnetic field strengths (1.5T and 3.0T) and scanner vendors.

**Results:**

In the test dataset, the T2-CET1 combined model outperformed both the independent CET1 and T2 models, achieving an AUC of 0.86, accuracy of 83.3%, sensitivity of 83.3%, and specificity of 83.8%. This compares favorably with the *CET1 model* (AUC: 0.80, accuracy: 73.3%, sensitivity: 80.0%, specificity: 66.7%) and the *T2 model* (AUC: 0.79, accuracy: 76.7%, sensitivity: 76.7%, specificity: 76.7%). The *combined model’s* superior performance extended to the external validation set, where its AUC (0.865) exceeded that of the *CET1 model* (0.765) and the *T2 model* (0.811). Performance varied by MRI field strength. For 1.5T systems, AUCs were 0.50 (CET1), 0.76 (T2), and 0.58 (combined). For 3.0T systems, the corresponding AUCs were 0.61, 0.83, and 0.56. Similarly, analysis by specific scanner model showed AUCs of 0.60 (CET1), 0.83 (T2), and 0.53 (combined) for one scanner, compared to 0.54, 0.84, and 0.52 for the other.

**Conclusions:**

Combining CET1 with T2 improves prediction performance for pituitary macroadenoma consistency. However, the T2 model demonstrates greater stability across different equipment than either the CET1 or combined models.

## Highlights

- Preoperative assessment of pituitary macroadenoma consistency can inform clinical management and predict surgical risk.
- The radiomics model using combined CET1 and T2 imaging sequences is superior to single-sequence models in predicting pituitary adenoma consistency.
- Compared to CET1-based or combined T2-CET1 models, the T2-based model exhibits lower variability across different field strengths and equipment manufacturers.

## 1 Introduction

Pituitary adenoma is the most common sellar tumor and the third most common intracranial tumor, accounting for approximately 10–25% of central nervous system tumors (1–4). Surgical resection, primarily via the transsphenoidal approach, represents the mainstay of treatment (5, 6). Accurately predicting the preoperative texture (non-fibrous vs. fibrous) of a pituitary macroadenoma is critical to optimizing the choice of surgical approach (7). For example, tumors predicted to be rigid or fibrotic may require a broader surgical approach (e.g., a double-nostril approach rather than a single-nostril approach) or advance planning of alternative approaches (e.g., a transcranial approach), especially in complex anatomical situations such as large tumors, invasion of the cavernous sinus, or significant expansion into the saddle (8–10). Radiomics quantifies imperceptible lesion characteristics by mathematically analyzing spatial distributions and pixel intensities within medical images. Recent studies have applied MRI radiomics to evaluate pituitary adenoma texture. For instance, Cuocolo et al. (11) developed an MR-based radiomics model predicting macroadenoma consistency, achieving an AUC of 0.99 and accuracy of 0.93. However, this study was limited by its small sample size, exclusive use of T2-weighted sequences, and reliance on unvalidated subjective intraoperative consistency assessments. Crucially, multi-sequence MRI typically provides richer diagnostic information (12–14), and contrast-enhanced T1-weighted (CE-T1) imaging—essential for pituitary adenoma diagnosis and differential diagnosis—remains unexplored in radiomics-based consistency prediction. This study aims to develop a more accurate predictive model for pituitary macroadenoma consistency using a larger patient cohort and multi-sequence MR images (including CE-T1), thereby optimizing surgical approach selection. Concurrently, we evaluate model stability under defined conditions.

## 2 Materials and methods

### 2.1 Patient data collection

**Figure 1:**
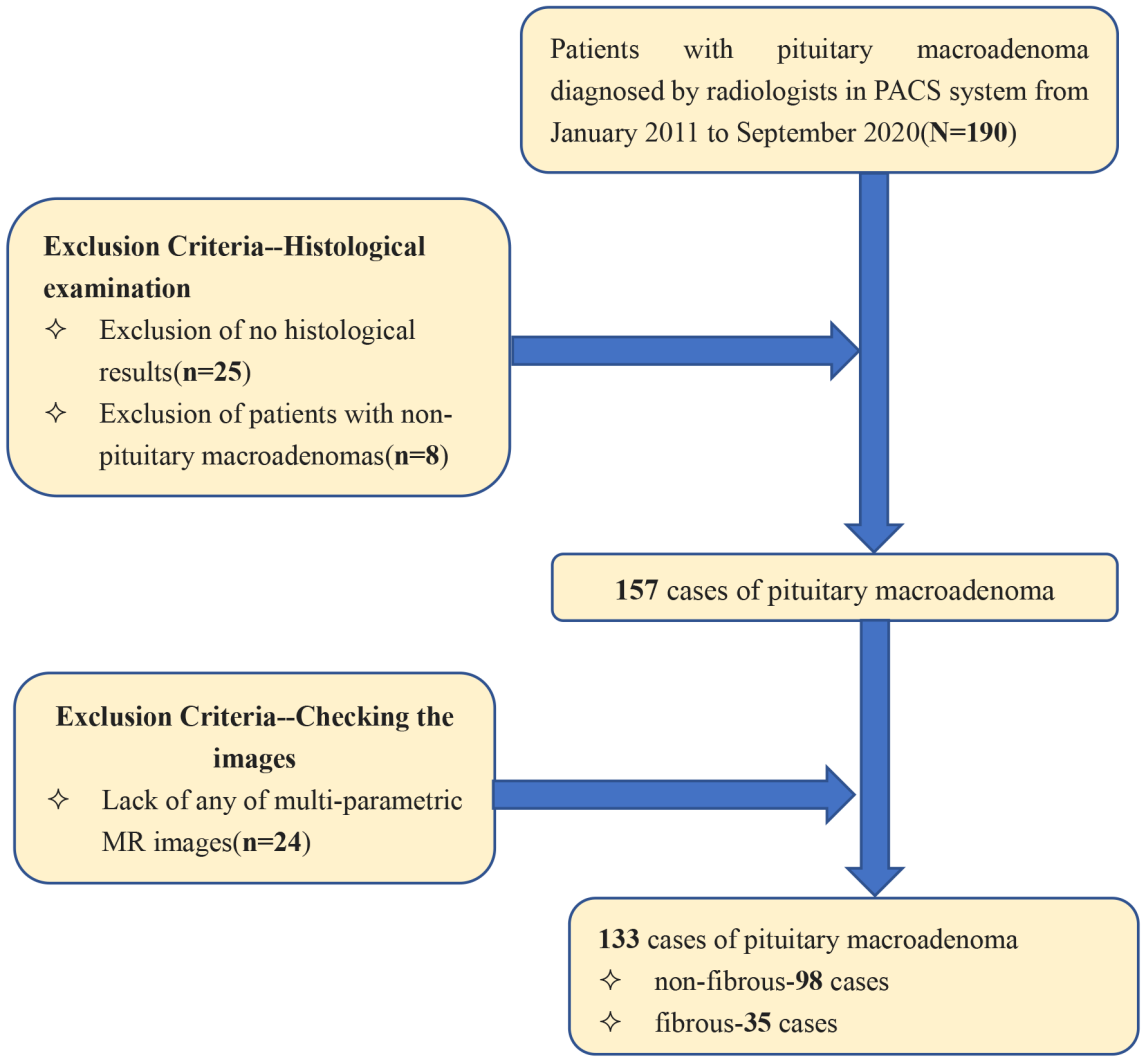
The patient screening process.

Patients diagnosed with pituitary macroadenoma between January 2011 and August 2020 were identified within the *Picture Archiving and Communication System* (PACS), yielding a total of 133 patients. Inclusion criteria were: (a) preoperative MR plain scan and contrast-enhanced examination with complete imaging data; (b) complete clinical data, including surgical records and postoperative pathological results; and (c) pituitary macroadenoma diameter ≥1 cm. Exclusion criteria were: (a) poor image quality inadequate for radiomic analysis; and (b) recurrent pituitary macroadenoma. The patient screening process is shown in **Figure 1**.

### 2.2 Study design

**Figure 2:**
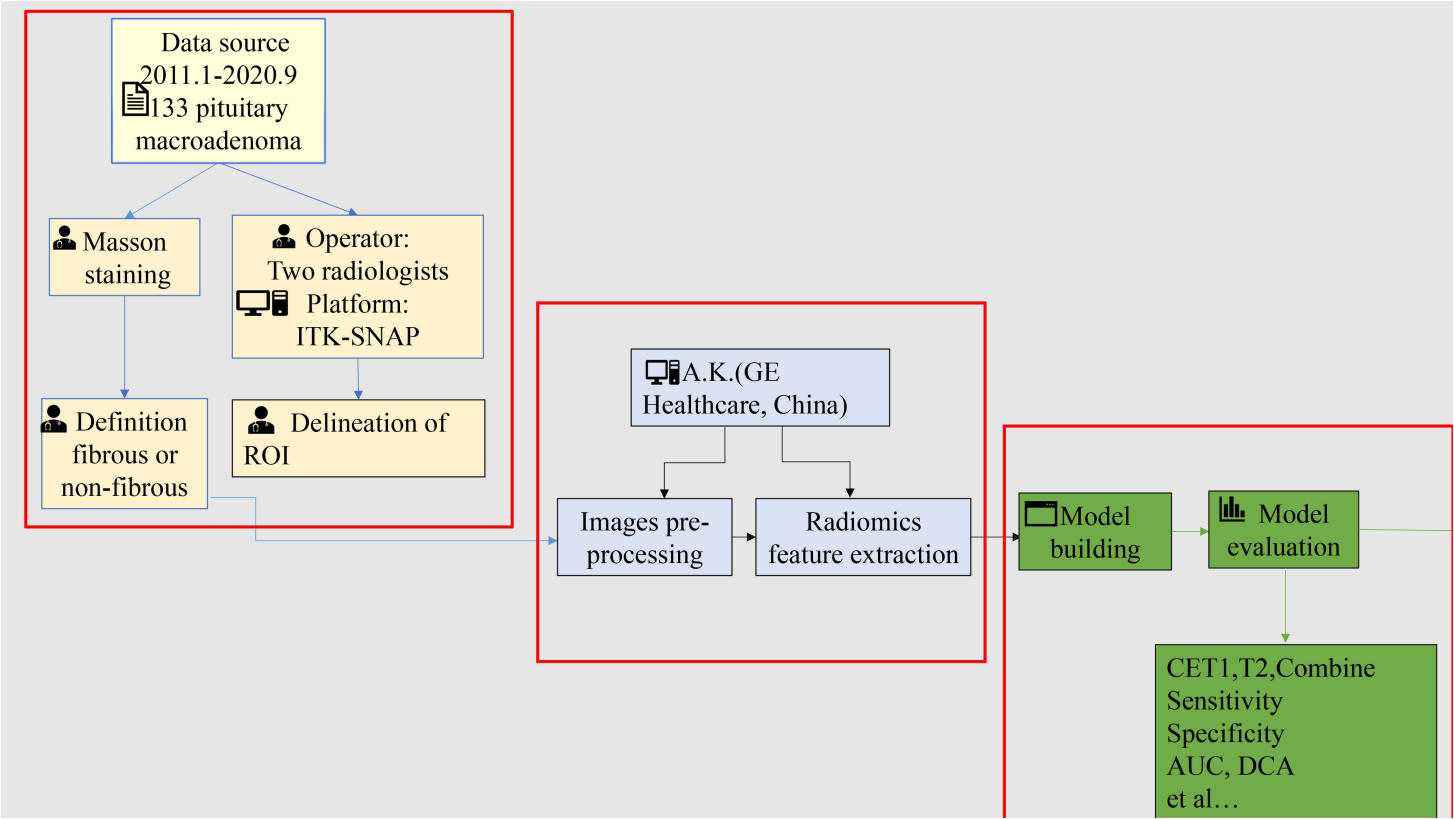
The study workflow.

The study workflow is shown in **Figure 2** and comprises four steps: (1) patient cohort enrollment and tumor segmentation, (2) feature extraction, (3) model construction and evaluation, and (4) stratified analysis based on equipment type and magnetic field strength.

### 2.3 Histologic study

In this study, tumor consistency was classified based on collagen expression levels measured in postoperative pathological sections. Sections were stained using the Masson trichrome method masson staining reagent purchased from Zhuhai Baso Biotechnology Co., Ltd., product code BA4079B). For each sample, five random fields of view were captured at ×200 magnification. The collagen-positive area within the tumor was quantified using ImageJ software (v1.8.0, National Institutes of Health) by measuring the blue-stained extracellular matrix area. Tumors were classified as non-fibrous if collagen constituted less than 15% of the total tumor area, and fibrous if collagen constituted 15% or more (15–18).

### 2.4 Image segmentation and radiomic feature extraction

CET1 images and T2 images of all patients were imported into ITK-SNAP (http://www.itksnap.org/, version: 3.8.0), and then two radiologists of different seniority(operated at an interval of 2 weeks) used a layer-by-layer manual outline of the lesion to generate a region of interest (ROI) (the delineation method was described in the **Supplementary Data Sheet 1**). The pre-processing, including Gaussian noise reduction, offset field correction, and histogram matching, was implemented on A.K. software (Artificial Intelligence Kit, A.K., version 3.3.0, GE Healthcare). After pre-processing, the image features were extracted using open-source Pyradiomics (19). Finally, 107 features were extracted from both the CET1 and the T2. Their detailed description is available in the online Pyradiomics documentation (https://pyradiomics.readthedocs.io/en/latest/features.html). Test of ICC for characteristics derived by two clinicians. Features with ICC > 0.75 were retained for further analyses.

### 2.5 Model building and evaluation

The enrolled patients were randomly allocated to a training set and a validation set in a 7:3 ratio. Using the ultimately selected feature set, prediction models based on T2, CE-T1, and combined T2 plus CE-T1 texture parameters were constructed via machine learning methods, including logistic regression, support vector machines (SVM), and decision trees. SMOTE was specifically employed to address the critical clinical problem of underrepresented difficult tumor cases in our dataset (20–22). Our approach directly addresses this gap by enhancing training data for edge cases where human experts typically struggle, aligning with clinical needs for improved difficult-case diagnostics. Patients were then randomly allocated to training and validation sets in a 7:3 ratio. After splitting the dataset into training and testing sets, the SMOTE was applied exclusively to the training set to balance the class distribution before model training. The testing set remained untouched throughout this process.To reduce information redundancy, radiomic features were filtered using a two-step process:

1. Correlation-based filtering: Features with an inter-feature correlation coefficient exceeding 0.90 were removed.
2. Low-variance filtering: Features exhibiting variance ≤ 0.1 were excluded. Subsequently, the Gradient Boosted Decision Tree (GBDT) algorithm was employed to further refine the feature set based on feature importance scores.

Logistic regression models were constructed using the final filtered features:

- Separate models for T2-weighted images and CET1-weighted images.
- A combined model integrating selected features from both image types.

For each model (individual or combined), a radiomics score (Radscore) was calculated using the formula:


Radscore = Intercept + ∑(β_i ∗ X_i)

where Intercept is the constant term, *β_i* represents the logistic regression coefficient for the *i*-th feature, and *X_i* is the feature value.

Model performance was evaluated using:

- Receiver Operating Characteristic (ROC) curves, reporting the Area Under the Curve (AUC), accuracy, sensitivity, specificity, positive predictive value (PPV), and negative predictive value (NPV).
- Calibration curves to assess agreement between predicted probabilities and actual outcomes.
- Decision Curve Analysis (DCA) to quantify the net clinical benefit across various threshold probabilities.

Finally, model stability was assessed via stratified analysis across different MRI field strengths (1.5T vs. 3.0T) and manufacturers (Siemens vs. Philips) the code was described in the **Supplementary Data Sheet 2** and **3**.

### 2.6 Statistical analysis

R (version 3.5.1) and Python (version 3.5.6) were used for feature selection, model building, and evaluation. To ensure model reproducibility, the inter-class correlation coefficient (ICC) was calculated to assess feature stability between two independent radiologists. Features with an ICC > 0.75 were retained, demonstrating relatively high inter-reader agreement in segmented tumor volume. Statistical significance was defined as a two-tailed *P* value below 0.05.

## 3 Results

### 3.1 Patient characteristics

We included a total of 133 patients: 35 in the hard group (16 male, 19 female; mean age 51.2 ± 12.4 years) and 98 in the soft group (46 male, 52 female; mean age 52.4 ± 13.2 years). There were no statistically significant differences in age or gender between the hard and soft groups (P > 0.05). In the external validation cohort, 40 patients were enrolled, comprising 20 patients with soft consistency (12 males and 8 females, mean age 54.3± 11.7 years) and 20 patients with hard consistency (12 males and 8 females, mean age 52.7 ± 14.1 years).

### 3.2 Training and validation of the radiomics-based machine learning

The intraclass correlation coefficient (ICC) values for radiomic features extracted from regions of interest (ROI) outlined by both operators exceeded 0.75. Following Spearman correlation analysis, 56 features from T2-weighted images and 19 features from contrast-enhanced T1-weighted (CET1) images were retained. Radscore calculation and detailed accuracy metrics are provided in the **Supplementary Materials**.

**Figure 3:**
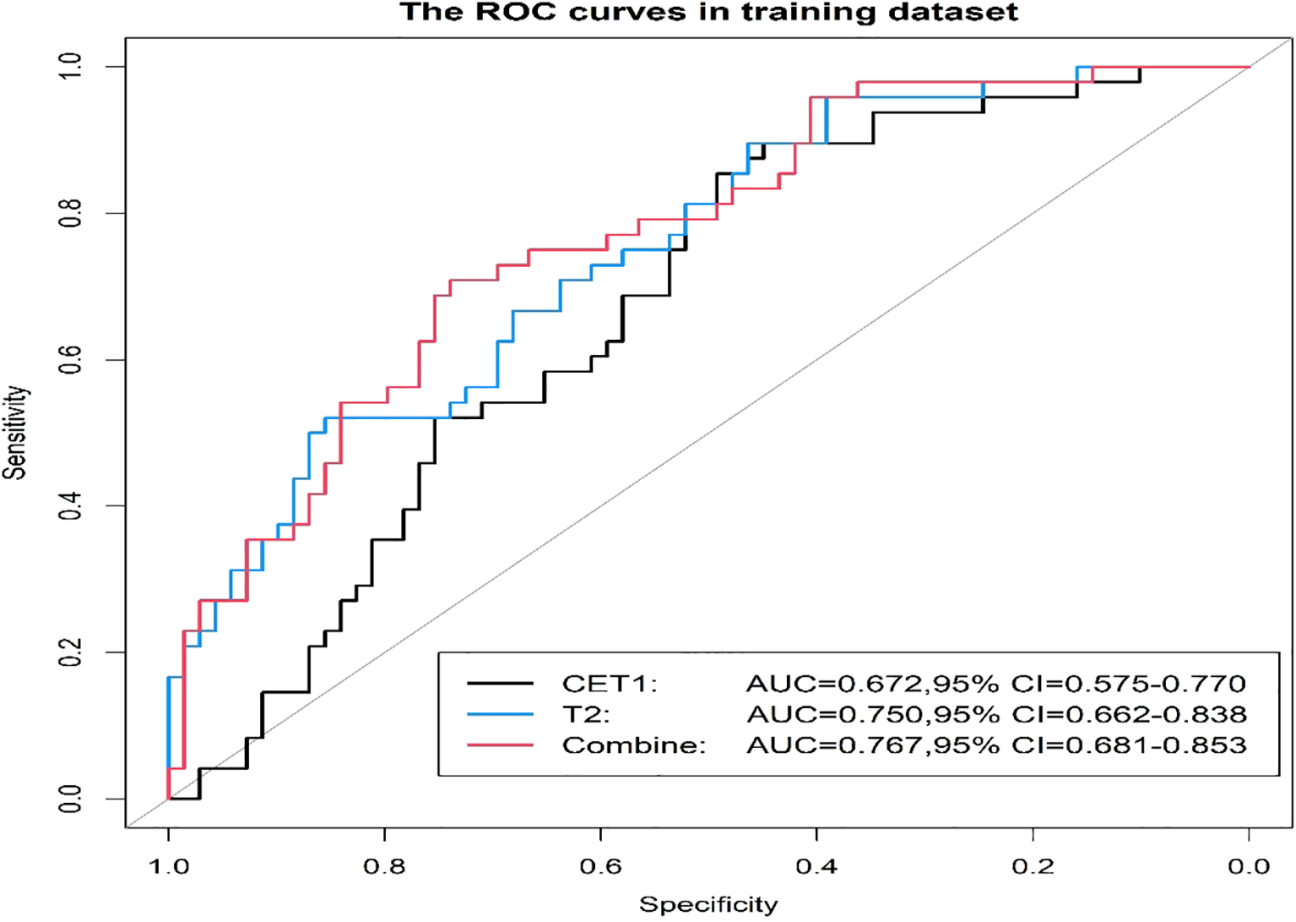
Three prediction models' ROC curves in the training set.

**Figure 4:**
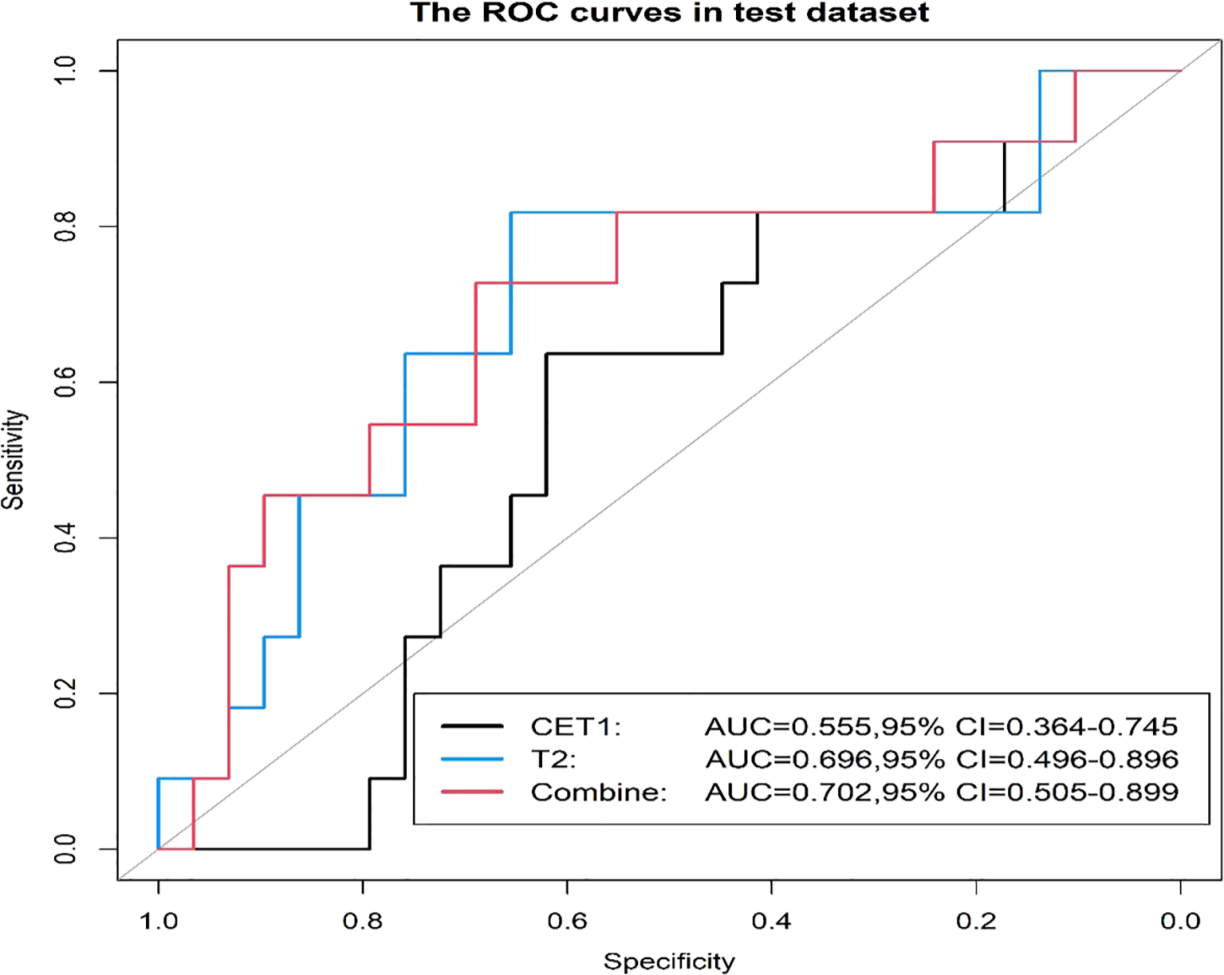
Three prediction models' ROC curves in the test set.

**Figure 5:**
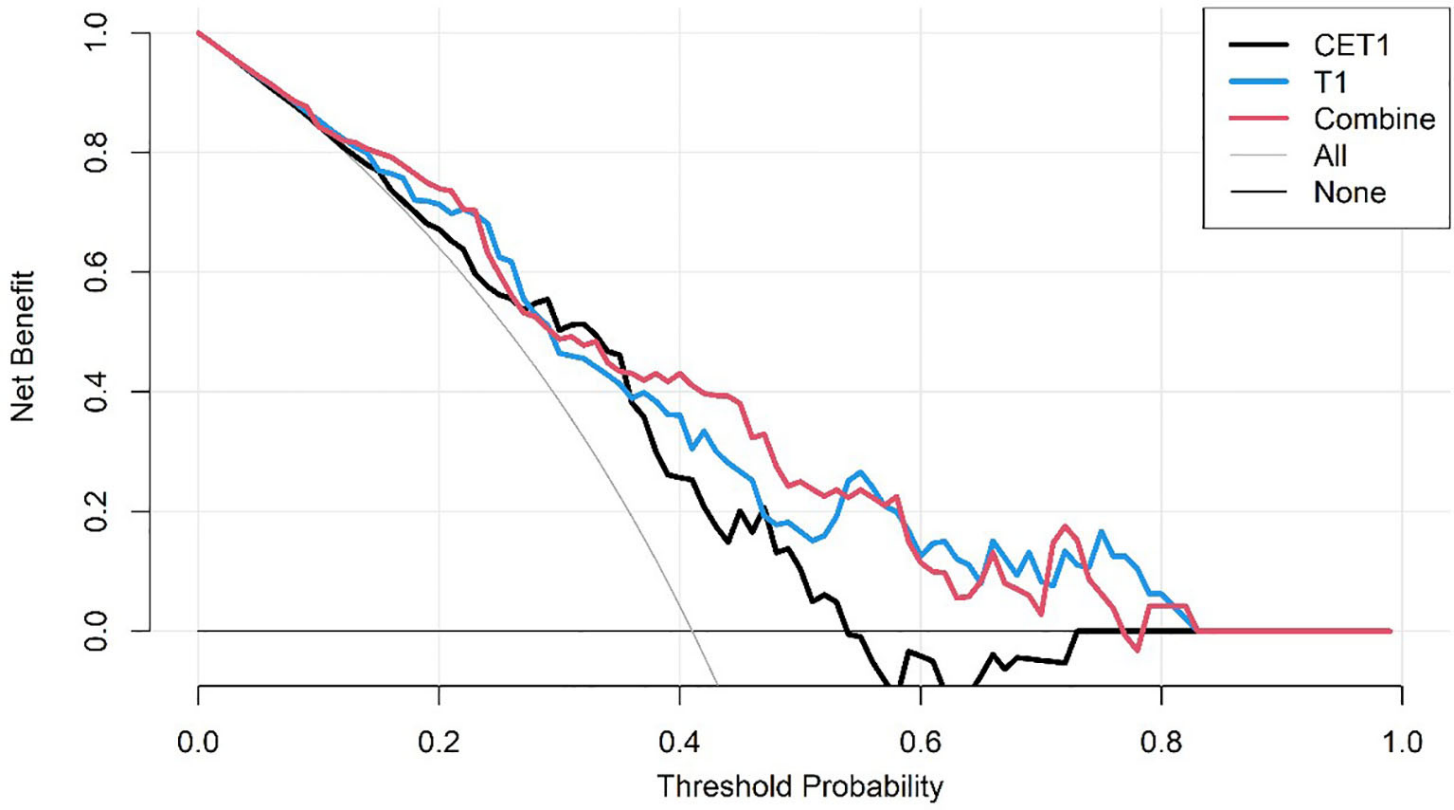
The DCA curves of the three predictive models on the training set.

**Figure 6:**
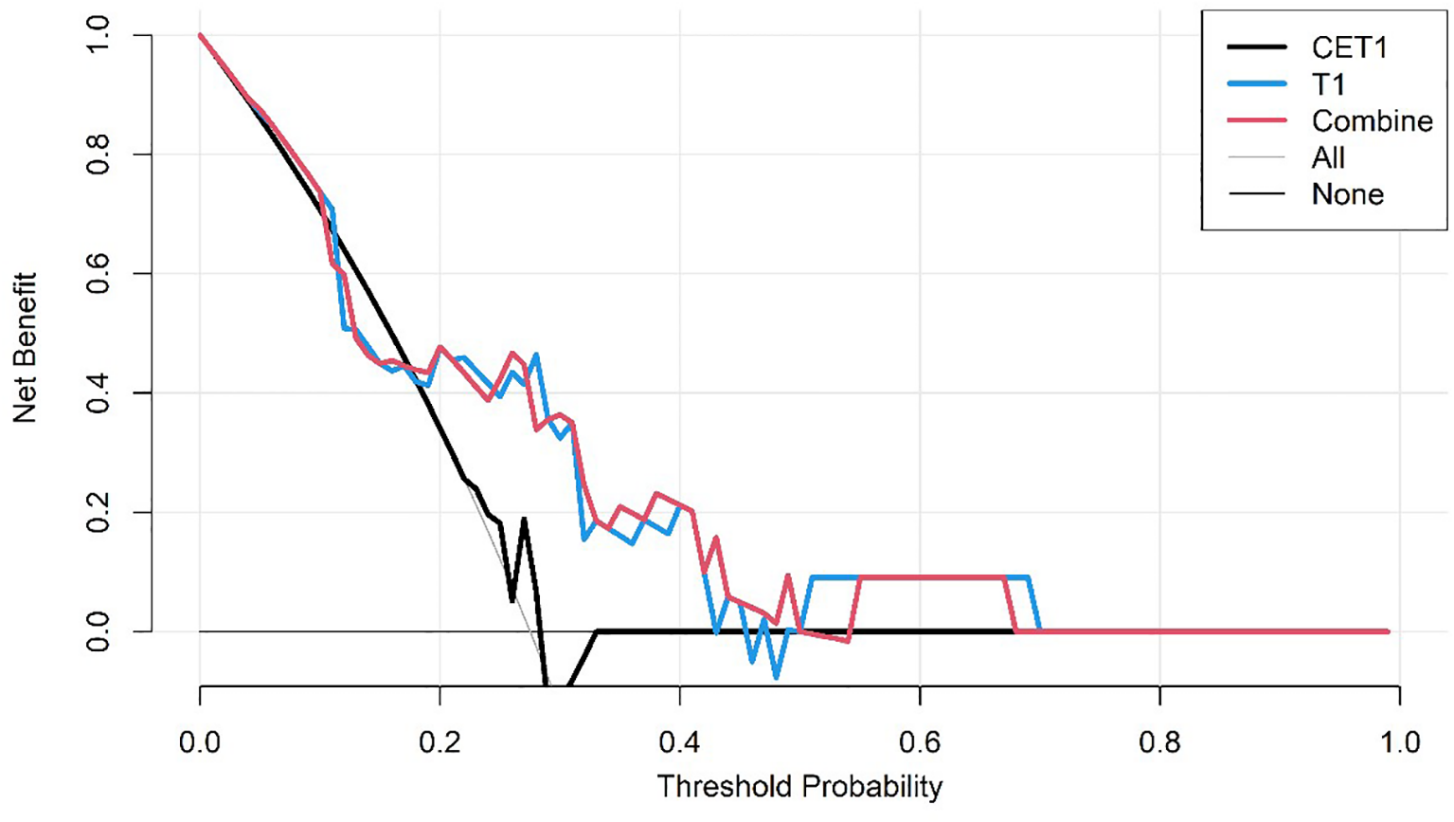
The DCA curves of the three predictive models on the test set.

Prior to applying SMOTE, the AUC values for the T2-based model, CE-T1-based model, and their combined model were 0.70, 0.56, and 0.70, respectively (**Figures 3**–**6**). Following the application of SMOTE, the combined model achieved an accuracy of 83.3%, sensitivity of 83.3%, specificity of 83.8%, and an AUC of 0.86. In comparison, the performance metrics for the individual models were:

- CET1 Model: Accuracy 73.3%, AUC 0.80, Sensitivity 80.0%, Specificity 66.7%
- T2 Model: Accuracy 76.7%, AUC 0.79, Sensitivity 76.7%, Specificity 76.7%

**Figure 7:**
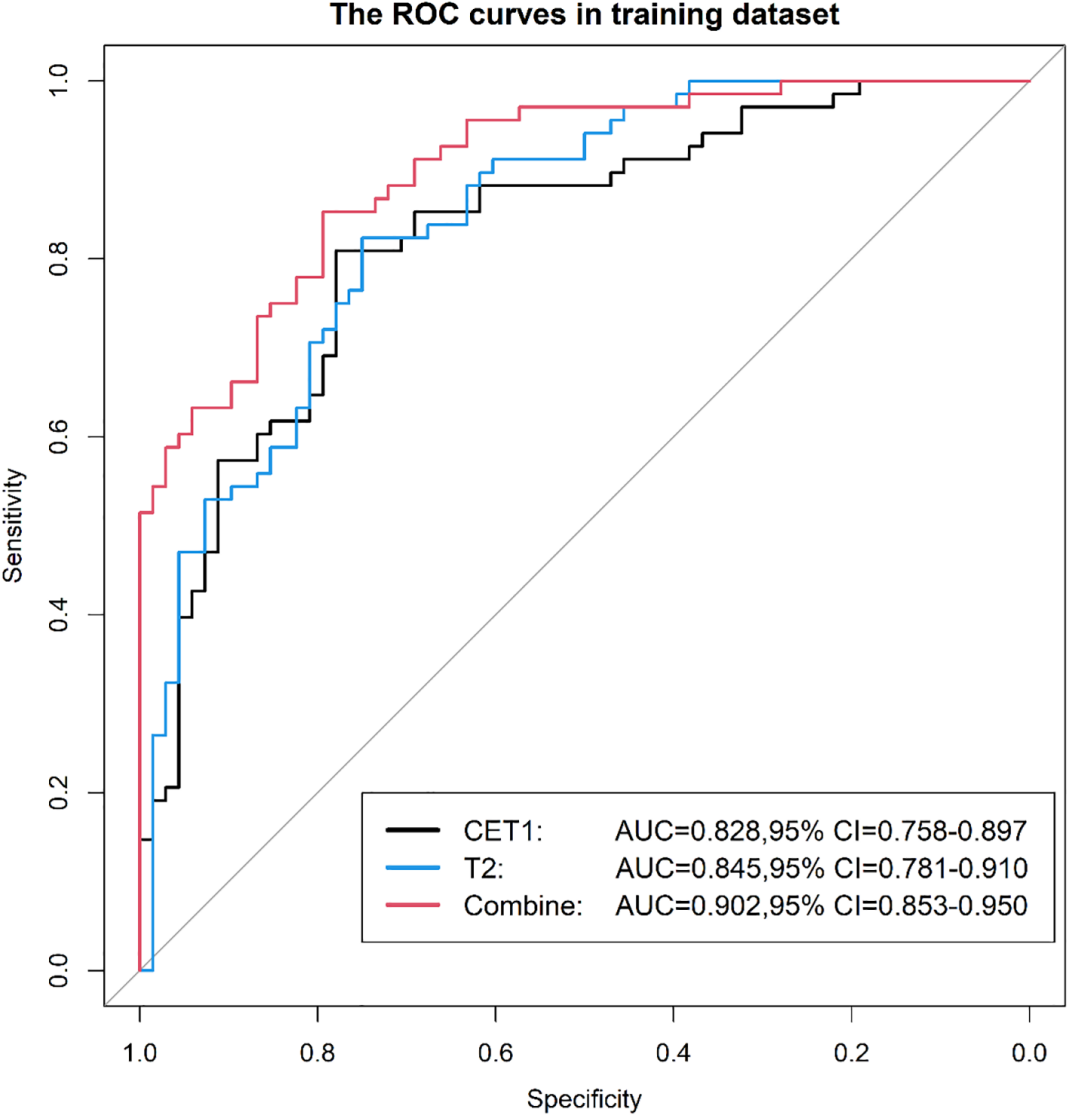
After applying SMOTE, the ROC curves of the three models on the training set.

**Figure 8:**
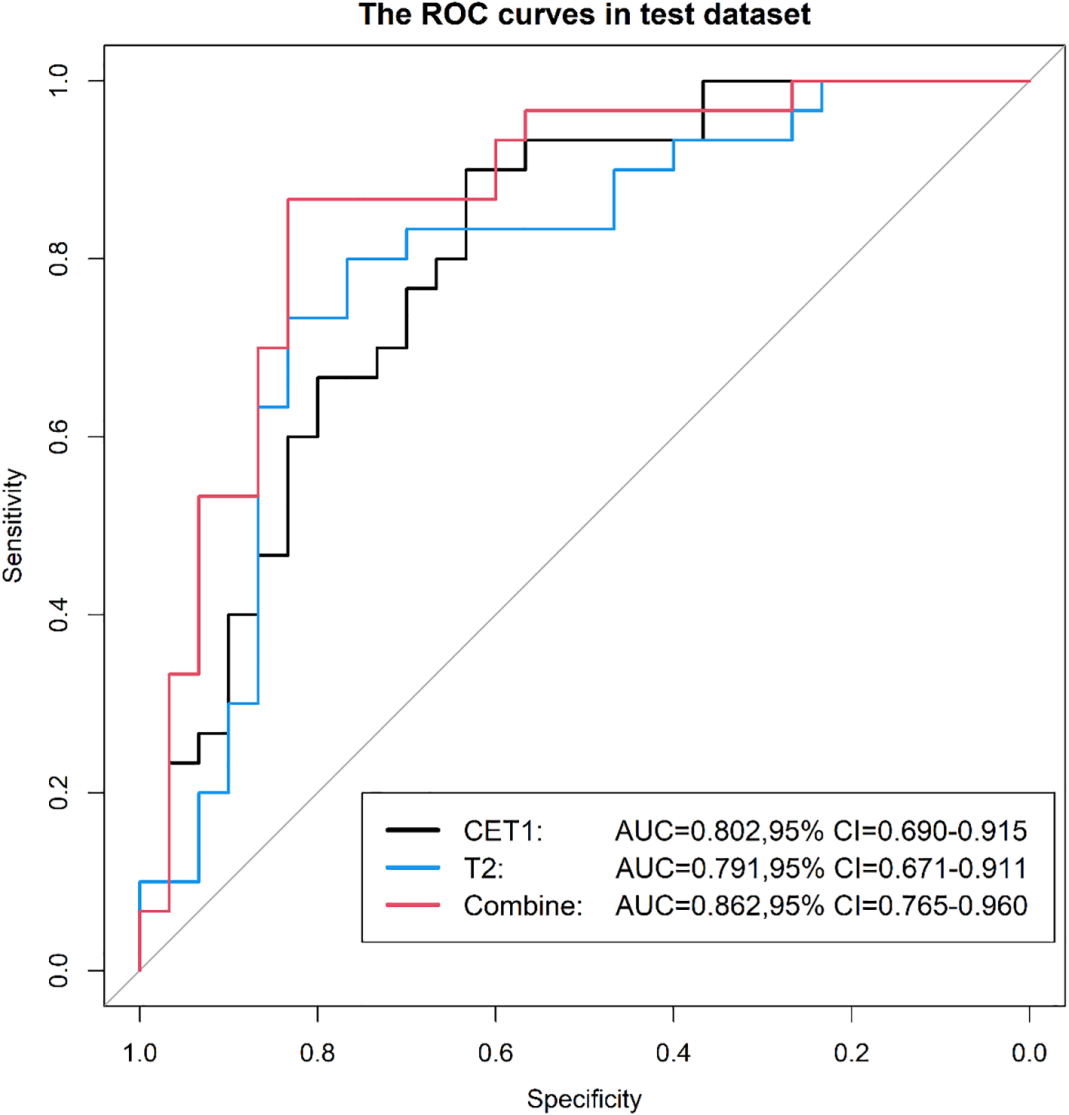
After applying SMOTE, the ROC curves of the three models on the test set.

**Figure 9:**
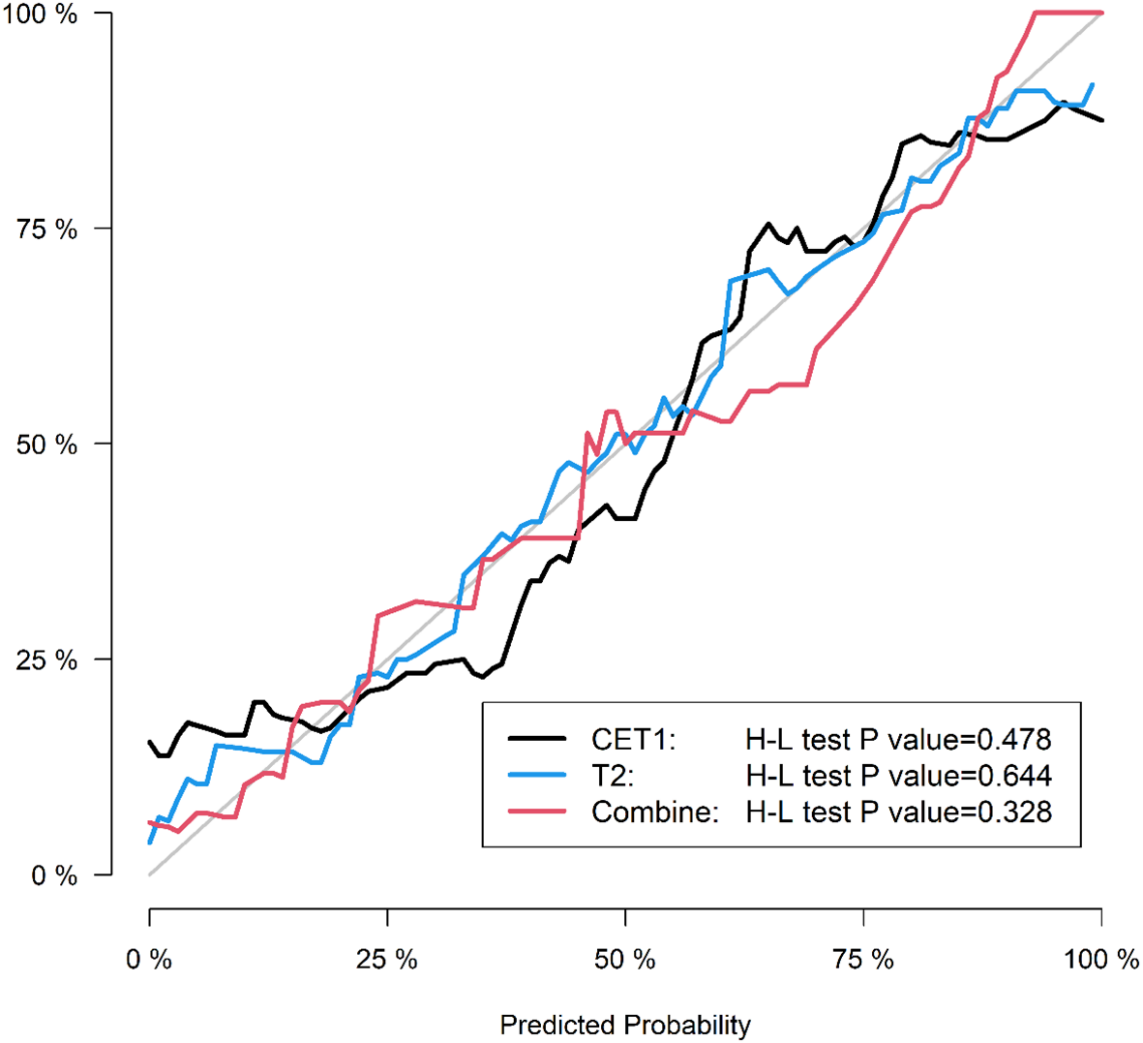
After applying SMOTE, the calibration curves of the three models on the training set.

**Figure 10:**
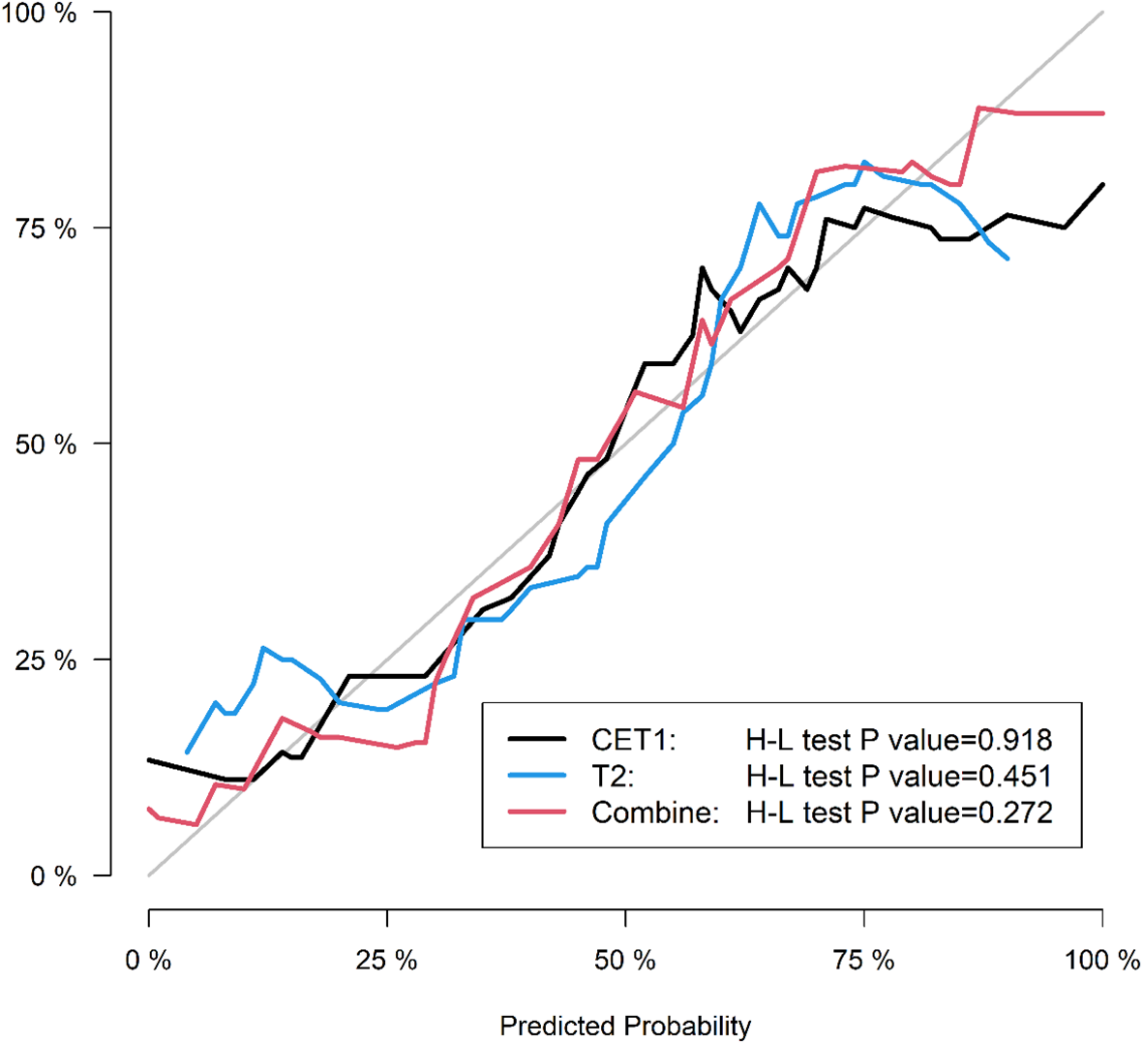
After applying SMOTE, the calibration curves of the three models on the test set.

**Figure 11:**
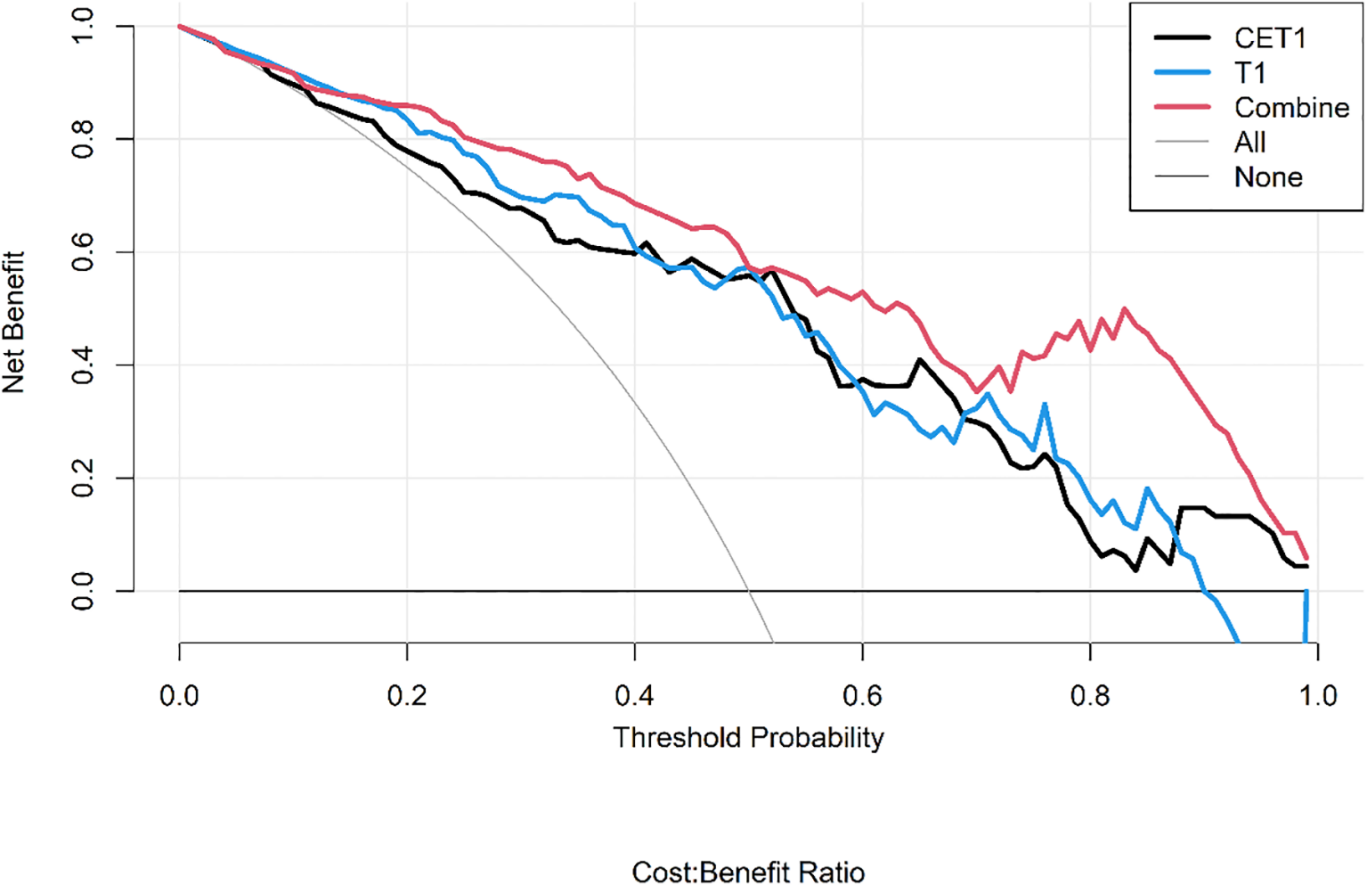
After applying SMOTE, the DCA curves of the three models on the training set.

**Figure 12:**
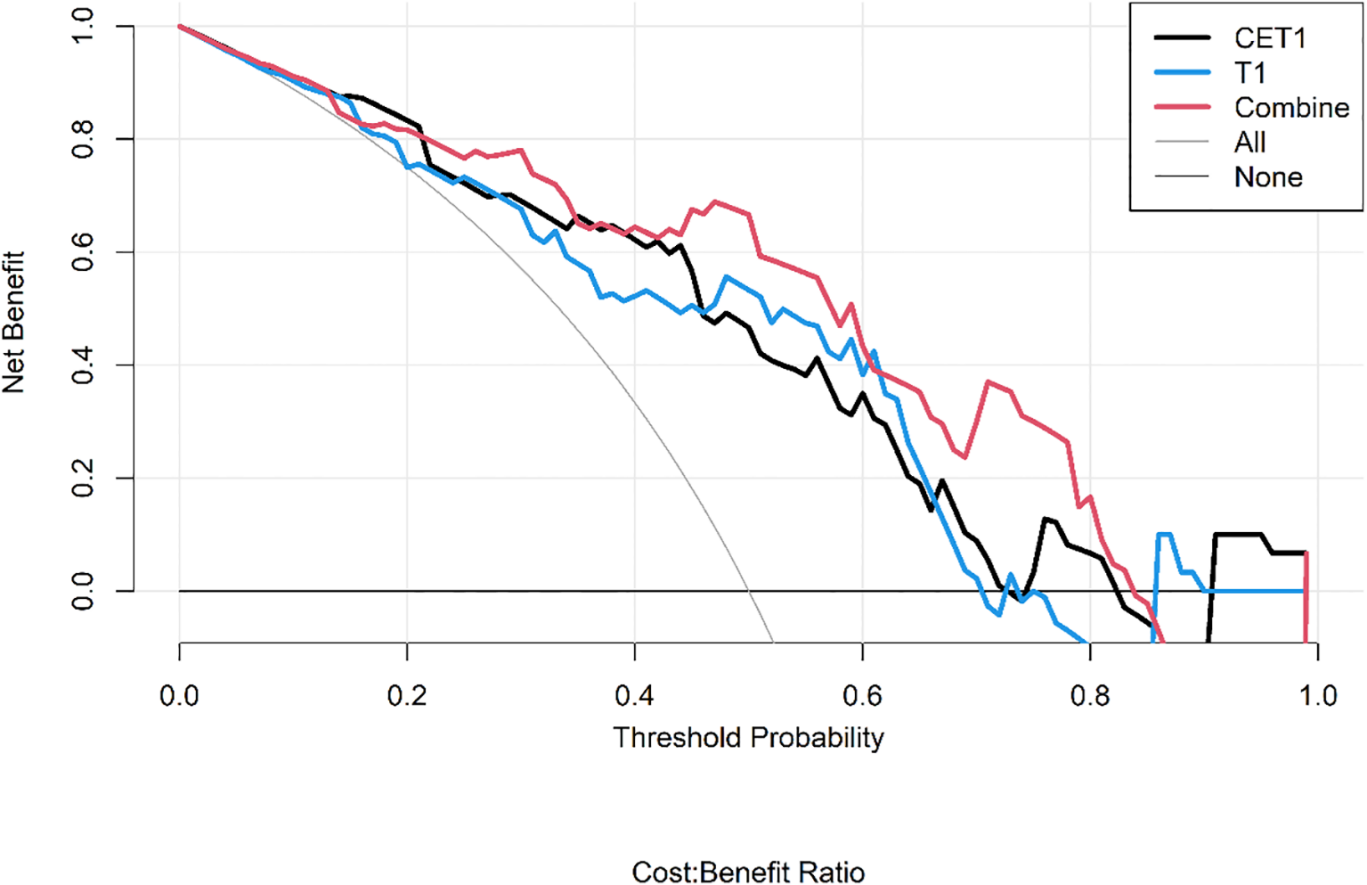
After applying SMOTE, the DCA curves of the three models on the test set.

Corresponding ROC curves are shown in **Figures 7**, **8**. Calibration curves (**Figures 9**, **10**) demonstrated good performance for all three models in both the training and validation cohorts. Decision curve analysis (DCA, **Figures 11**, **12**) indicated that the combined model yielded the highest net benefit across nearly all threshold probabilities.

**Figure 13:**
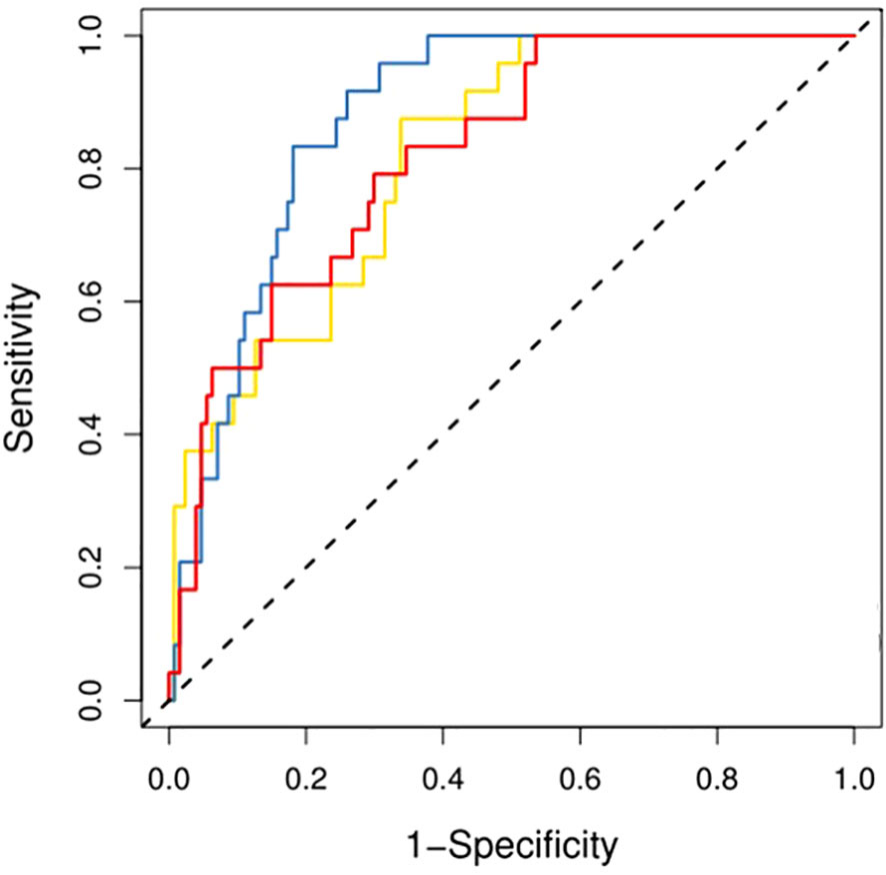
The ROC curves of the three models on the external validation set.

On the external validation set, the combined model maintained superior performance with an AUC of 0.865, outperforming the CET1 model (AUC 0.765) and the T2 model (AUC 0.811). The ROC curves for external validation are presented in **Figure 13**.

### 3.3 Evaluation of radiomics model in different equipment (different field strengths and different vendor)

**Figure 14:**
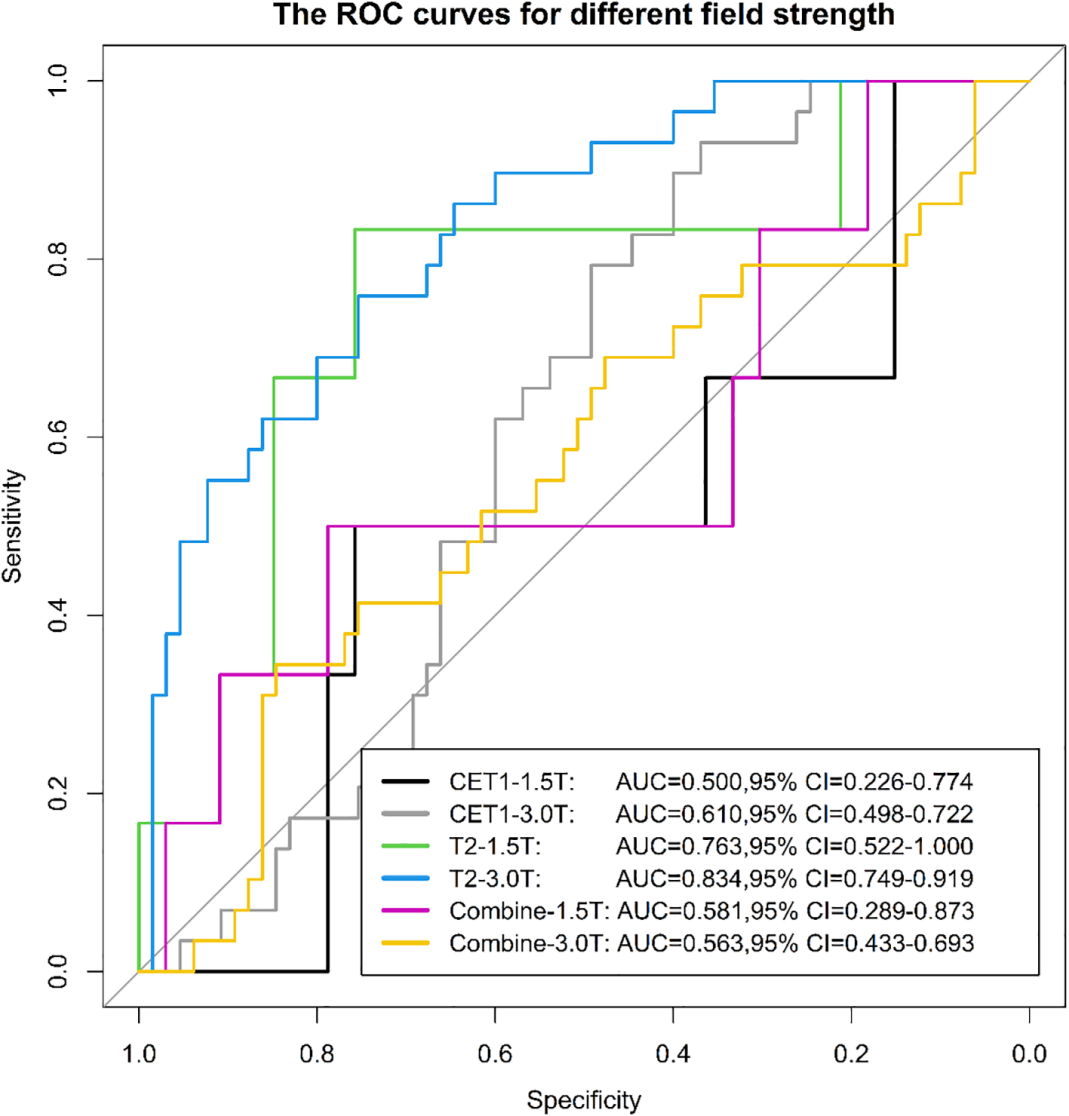
The ROC curves for different field strength.

**Figure 15:**
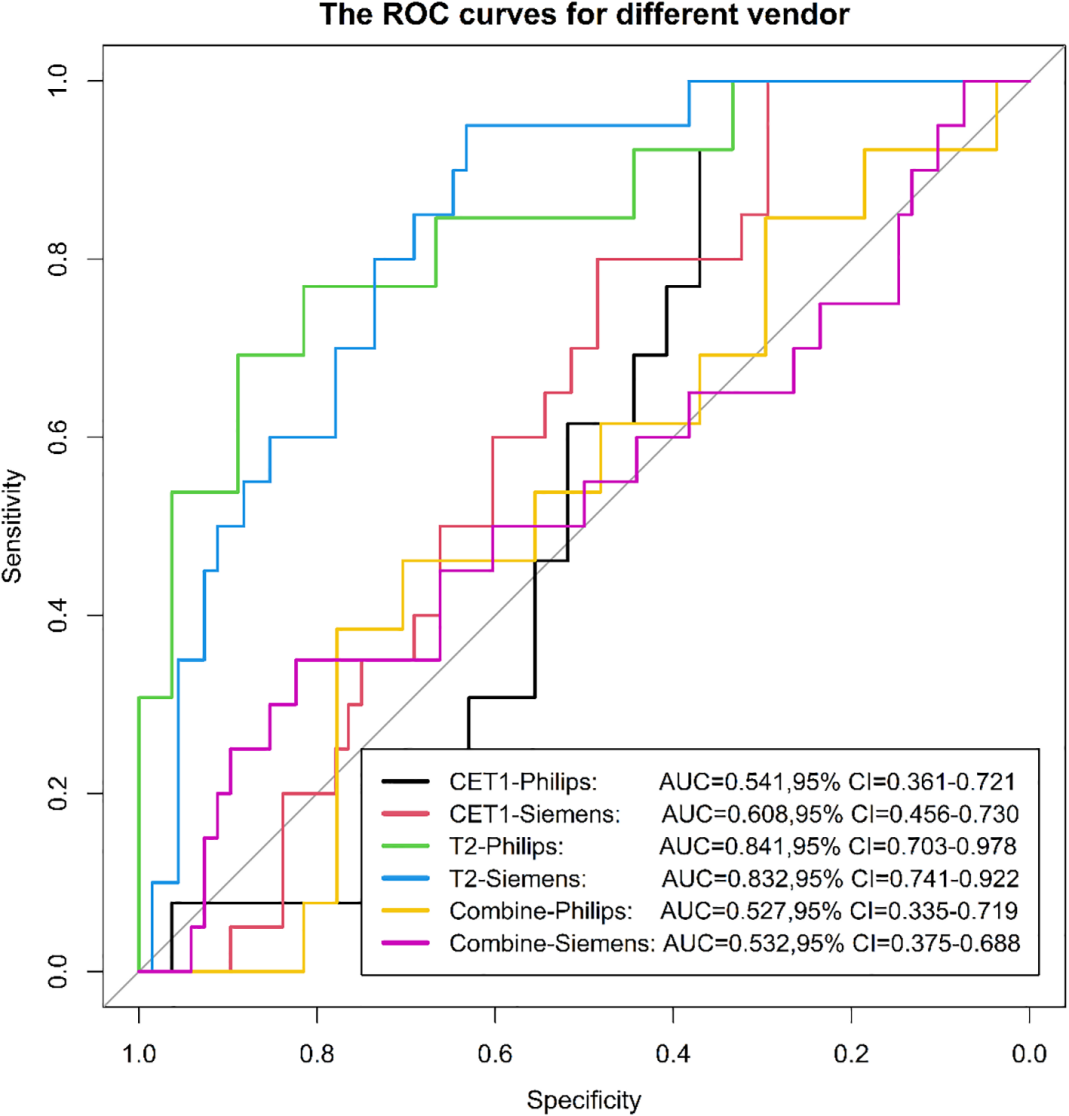
The ROC curves for different vendor.

Among 133 included patients, 128 were analyzed by scanner vendor (GE Healthcare [n=5] was excluded from stratified analysis due to small sample size). Scans were performed using Philips Healthcare (all 3.0T; n=39) or Siemens Healthcare equipment (1.5T: n=34; 3.0T: n=55). Results demonstrated that the model derived from T2-weighted images alone exhibited greater stability across different scanner types (both vendors and field strengths) compared to models using CET1-weighted images alone or combined T2 and CET1 images (**Figures 14**, **15**).

## 4 Discussion

In our study, we first developed models to predict pituitary macroadenoma consistency using CET1 images and T2 images separately, then created a combined model using both sequences. The combined model outperformed models based on either sequence alone. However, further stratified analysis revealed that the model built solely on T2 images demonstrated greater stability than either the CET1-only model or the combined model.

The radiomic model combining CET1 and T2 images for preoperative prediction of pituitary macroadenoma consistency achieved an accuracy of 0.833 and an AUC of 0.862. In contrast, prediction models developed by Cuocolo et al. (11) (using T2 images) and Su et al. (23) (using DWI, b=2000 s/mm²) reported higher AUCs of 0.99 and 0.91, respectively, indicating superior diagnostic performance to our model. Several factors may explain this performance difference. First, our study included a larger cohort (133 patients) compared to Cuocolo et al. (89 patients) and Su et al. (50 patients). Second, while the prior studies relied on subjective intraoperative surgeon assessment of tumor consistency (despite initial training), our methodology used Masson staining of pathological sections for classification (18), providing a more objective and accurate standard. Furthermore, regarding imaging sequences, CET1 may offer a more realistic depiction of pituitary macroadenomas than DWI.

We evaluated the stability of three models (T2, CET1, combined) across different field strengths (1.5T and 3.0T) and MRI vendors. The T2-based model demonstrated significantly higher stability than either the CET1 or *combined* models. We posit that two interrelated factors—*field strength-dependent signal heterogeneity* and *vendor-/field strength-dependent differences in contrast enhancement behavior*—likely contributed to this phenomenon, particularly affecting the integrated nature of the combined model.

### 4.1 Field strength-dependent signal heterogeneity

Fundamental physical differences between 1.5T and 3.0T scanners significantly impact signal-to-noise ratio (SNR), contrast-to-noise ratio (CNR), and spatial resolution. While 3.0T generally offers higher SNR enabling finer texture resolution (24), it also introduces greater susceptibility artifacts, spatial inhomogeneity, and B1 field inhomogeneity. Radiomic features, especially texture descriptors (e.g., GLCM, GLRLM, GLSZM), are inherently sensitive to these variations in image acquisition physics (25, 26). This non-biological, acquisition-driven heterogeneity disrupts the stability and reproducibility of radiomic features across field strengths (27). Consequently, the model weights optimized on the training data (potentially dominated by one field strength) may misrepresent feature-tumor biology relationships in the other field strength subgroup, leading to performance degradation in stratified testing (28).

### 4.2 Vendor/field strength-dependent contrast enhancement behavior

Gadolinium-based contrast enhancement kinetics and appearance in pituitary macroadenomas are influenced by complex interactions between tumor biology (e.g., vascularity, permeability) and technical factors. Crucially, relaxivity (R1) of gadolinium chelates is field-strength dependent (29), and vendor-specific implementations of pulse sequences (e.g., saturation pulses, flip angle optimization, parallel imaging) further modulate signal dynamics during contrast uptake (30). Our combined model relies heavily on radiomic features extracted from post-contrast T1-weighted sequences (e.g., firstorder_Minimum, firstorder_MeanAbsolute, glcm_JointEntropy), which encode information about enhancement intensity and heterogeneity. Systematic differences in the *apparent* enhancement patterns—driven by field strength (e.g., different T1-weighting at 1.5T vs 3.0T) and vendor-specific image reconstruction algorithms—can alter these feature values without reflecting true biological differences (31). The combined model, seeking synergy between clinical factors (e.g., hormone status) and radiomic phenotypes, may inadvertently learn spurious correlations between clinical variables and these acquisition-biased enhancement features. When applied to data from a different scanner type or field strength, these learned associations fail, degrading model performance (32). The *combined model*, however, aims to leverage complementary information. If the radiomic component introduces unstable, acquisition-dependent signals (as described above), the integration process can amplify noise rather than biological signal in heterogeneous test sets (33). This underscores the paradox that combining data sources can *reduce* robustness if one source (here, radiomics) lacks harmonization across acquisition platforms (34).

This methodological choice carries substantial clinical implications: Firstly, we adopted a more objective method for evaluating the texture of pituitary macroadenomas, defining it by the expression level of collagen within the tumor tissue. This represents the first time collagen quantification has been used to define texture in radiomics model predictions for pituitary macroadenomas. Secondly, we utilized contrast-enhanced sequences for the first time in this context. While these sequences are commonplace in the routine imaging evaluation of pituitary macroadenomas, their application in radiomics texture prediction is novel. It is well-established that fibrous components within tumors exhibit delayed enhancement on contrast-enhanced images. Previously employed sequences, such as T2-weighted images (T2WI) and diffusion-weighted imaging (DWI), fail to adequately capture the presence of these fibrous elements. Furthermore, contrast-enhanced sequences effectively depict necrotic and cystic components within the tumor. Undoubtedly, these components also significantly influence pituitary macroadenoma texture. In addition, we performed a first-ever stratified analysis of the constructed imaging model. Given that different hospitals employ varying imaging equipment for pituitary macroadenoma examinations, conducting this stratified analysis helps identify more stable models. This approach enhances the reproducibility of the radiomics model and facilitates its practical application in real-world clinical settings.

The preoperative prediction of tumor consistency holds significant potential to refine surgical strategies, particularly in selecting optimal operative corridors. Our radiomic model may directly influence decision-making in the following clinical scenarios:

1. Endonasal Approach Selection (Mononostril vs. Binostril): Predicted non-Fibrous Tumors: A mononostril transsphenoidal approach is often sufficient for predominantly soft lesions. These tumors can be efficiently aspirated or curetted through a single naris, minimizing nasal trauma and reducing operative time. Predicted Fibrous Tumors: Binostril endoscopic approaches become preferable when firm consistency is anticipated. The wider exposure facilitates bimanual microdissection, enhances instrument maneuverability for piecemeal resection, and allows safer dissection of adherent tumor capsules from neurovascular structures (e.g., optic apparatus, cavernous sinus). Failure to anticipate firm consistency via a mononostril corridor may lead to incomplete resection or excessive traction injury.

2. Consideration of Transcranial Access: Predicted firm consistency combined with specific anatomical factors may warrant transcranial approaches (e.g., pterional, subfrontal): For tumors exhibiting significant suprasellar extension (>3cm), a potential fibrous capsule may create adhesions tethering the tumor to critical structures like the optic chiasm or anterior cerebral arteries. In these cases, a transcranial approach enables direct visualization and sharp dissection of these adherent interfaces. Similarly, in Knosp Grade 3–4 tumors invading the cavernous sinus, a firm consistency elevates the risk of carotid artery injury during transsphenoidal dissection. Here, a transcranial or combined approach provides superior control for managing the lateral compartments.

The present study also has certain limitations. Firstly, it was a single-center retrospective study and the sample size included was only 133. Secondly, there was a large difference in the number of patients with non-fibrous and fibrous consistency, but this was consistent with the actual clinical situation and the epidemiology of pituitary macroadenoma. Last but at least, some of the cases we included have a long history, and the degradation of collagen in the pathological tissue may affect the qualitative judgment.

In conclusion, in the prediction of the consistency of pituitary macroadenomas, radiomics models based on CET1 images combined with T2 images have higher diagnostic efficacy than models constructed from independent images. However, the model constructed from independent T2 images was more stable across different field strengths and vendors.

## Data Availability

The raw data supporting the conclusions of this article will be made available by the authors, without undue reservation.
