# Supplementary material for: MR radiomics in assessment of consistency of pituitary macroadenoma: can T1-weighted contrast enhanced image improve diagnostic performance of T2-weighted image?
Source: Front Oncol. 2025 Sep 3;15:1539432. doi: 10.3389/fonc.2025.1539432 (PMC12440780; doi:10.3389/fonc.2025.1539432)
Supplement: Supplementary file 1 [file DataSheet1.docx]

**Supplementary Material 1.** MR images with segmentation of one example patient. Upper row: CET1 and manual segmentation image(*a, b, c*). Bottom row: T2 and manual segmentation image(*d, e, f*)


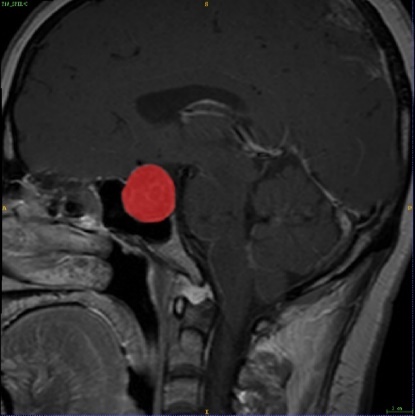

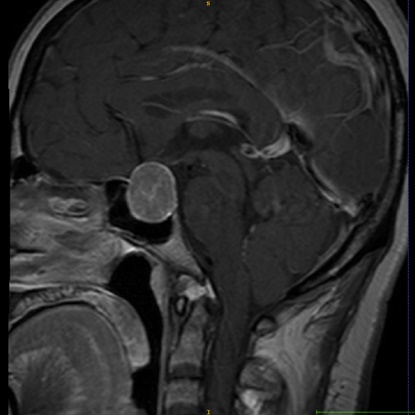

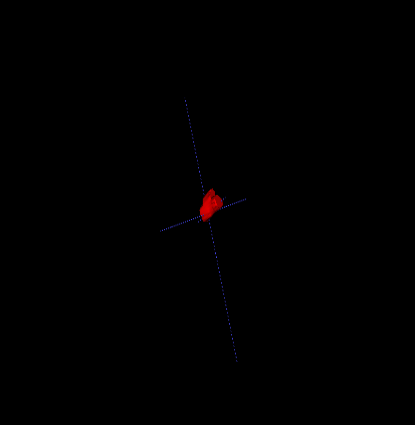


*a b c*


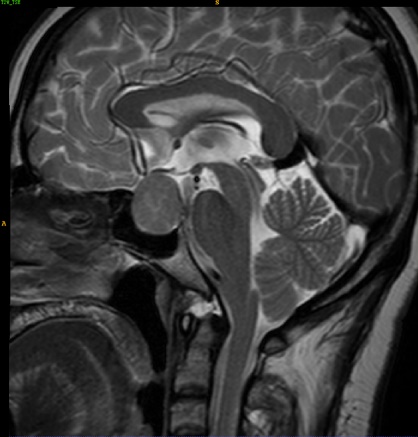

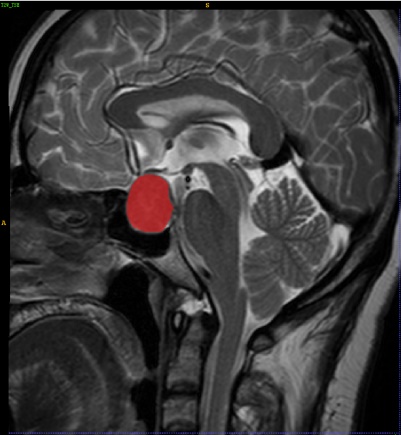

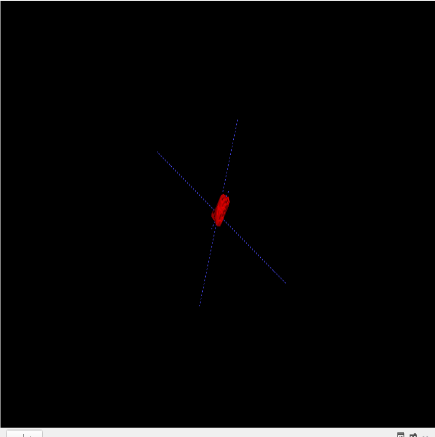


*d e f*

**Supplementary Material2.**The table respectively represent the evaluation of the logical model of T2, CET1, and combining in the training group and the validation group.

| Model | Item | Train | validation |
| --- | --- | --- | --- |
| *T2* | Accuracy | 0.779 | 0.767 |
|  | f1_score | 0.786 | 0.767 |
|  | AUC | 0.845 (0.789, 0.898) | 0.791 (0.684, 0.889) |
|  | Sensitivity | 0.809 | 0.767 |
|  | Specificity | 0.75 | 0.767 |
|  | positive prediction | 0.764 | 0.767 |
|  | negative prediction | 0.797 | 0.767 |
|  | positive llr | 3.235 | 3.286 |
|  | negatice llr | 0.255 | 0.304 |
| *CET1* | Accuracy | 0.787 | 0.733 |
|  | f1_score | 0.788 | 0.75 |
|  | AUC | 0.828 (0.767, 0.886) | 0.802 (0.706, 0.89) |
|  | Sensitivity | 0.794 | 0.8 |
|  | Specificity | 0.779 | 0.667 |
|  | positive prediction | 0.783 | 0.706 |
|  | negative prediction | 0.791 | 0.769 |
|  | positive llr | 3.6 | 2.4 |
|  | negatice llr | 0.264 | 0.3 |
| *Combining* | Accuracy | 0.816 | 0.833 |
|  | f1_score | 0.82 | 0.833 |
|  | AUC | 0.902 (0.859, 0.94) | 0.862 (0.779, 0.941) |
|  | Sensitivity | 0.838 | 0.833 |
|  | Specificity | 0.794 | 0.833 |
|  | positive prediction | 0.803 | 0.833 |
|  | negative prediction | 0.831 | 0.833 |
|  | positive llr | 4.071 | 5.0 |
|  | negatice llr | 0.204 | 0.2 |

**Supplementary Material 3.** Tables *a*, *b*, and *c* respectively represent the results of multi-factor logistic regression analysis of the texture features of the *T2, CET1*, and *combine* models

Table *a*

| variables | Coef. | Std.Err. | z | P>\|z\| | [0.025 | 0.975] |
| --- | --- | --- | --- | --- | --- | --- |
| intercept | -0.1830 | 0.2295 | -0.7974 | 0.4252 | -0.6329 | 0.2669 |
| original_firstorder_Energy | -0.3624 | 0.3748 | -0.9670 | 0.3336 | -1.0969 | 0.3722 |
| original_firstorder_Minimum | 0.1195 | 0.3049 | 0.3920 | 0.6951 | -0.4781 | 0.7171 |
| original_firstorder_Skewness | -0.1336 | 0.3929 | -0.3401 | 0.7338 | -0.9038 | 0.6365 |
| original_glcm_Contrast | -1.0393 | 0.5744 | -1.8092 | 0.0704 | -2.1651 | 0.0866 |
| original_glcm_Correlation | -0.6420 | 0.6132 | -1.0469 | 0.2951 | -1.8438 | 0.5599 |
| original_glcm_Imc1 | -1.0417 | 0.7041 | -1.4794 | 0.1390 | -2.4217 | 0.3384 |
| original_glcm_MaximumProbability | -0.0048 | 0.5315 | -0.0090 | 0.9928 | -1.0466 | 1.0370 |
| original_gldm_DependenceNonUniformityNormalized | 1.8214 | 0.7154 | 2.5460 | 0.0109 | 0.4192 | 3.2236 |
| original_gldm_LargeDependenceHighGrayLevelEmphasis | 1.2162 | 0.4560 | 2.6669 | 0.0077 | 0.3224 | 2.1100 |
| original_glrlm_LongRunLowGrayLevelEmphasis | -0.0083 | 0.5542 | -0.0150 | 0.9881 | -1.0945 | 1.0779 |
| original_glszm_GrayLevelNonUniformity | 1.0921 | 0.4273 | 2.5559 | 0.0106 | 0.2546 | 1.9296 |
| original_glszm_LargeAreaHighGrayLevelEmphasis | 0.0283 | 0.3145 | 0.0900 | 0.9283 | -0.5881 | 0.6447 |
| original_glszm_LowGrayLevelZoneEmphasis | 0.0414 | 0.5483 | 0.0755 | 0.9398 | -1.0333 | 1.1161 |
| original_glszm_SmallAreaEmphasis | -1.4669 | 0.4447 | -3.2984 | 0.0010 | -2.3386 | -0.5952 |
| original_glszm_SmallAreaLowGrayLevelEmphasis | -0.1827 | 0.4560 | -0.4008 | 0.6886 | -1.0765 | 0.7110 |
| original_glszm_ZoneEntropy | -1.3275 | 0.5658 | -2.3463 | 0.0190 | -2.4365 | -0.2186 |
| original_ngtdm_Strength | 1.5798 | 0.4231 | 3.7343 | 0.0002 | 0.7507 | 2.4090 |
| original_shape_Elongation | 0.2971 | 0.3855 | 0.7708 | 0.4408 | -0.4584 | 1.0526 |
| original_shape_Flatness | 0.5073 | 0.4150 | 1.2223 | 0.2216 | -0.3062 | 1.3207 |
| original_shape_Maximum2DDiameterColumn | 0.1340 | 0.4573 | 0.2930 | 0.7695 | -0.7624 | 1.0304 |
| original_shape_Sphericity | -0.5611 | 0.3211 | -1.7472 | 0.0806 | -1.1905 | 0.0683 |

Table *b*

| variables | Coef. | Std.Err. | z | P>\|z\| | [0.025 | 0.975] |
| --- | --- | --- | --- | --- | --- | --- |
| intercept | -0.0857 | 0.2181 | -0.3931 | 0.6942 | -0.5131 | 0.3417 |
| original_firstorder_Maximum | -1.9846 | 0.8568 | -2.3163 | 0.0205 | -3.6638 | -0.3053 |
| original_firstorder_Minimum | 0.5740 | 0.4161 | 1.3796 | 0.1677 | -0.2415 | 1.3895 |
| original_firstorder_Skewness | 2.0524 | 0.5602 | 3.6638 | 0.0002 | 0.9544 | 3.1503 |
| original_firstorder_TotalEnergy | 0.4558 | 0.4294 | 1.0613 | 0.2885 | -0.3859 | 1.2975 |
| original_glcm_ClusterShade | -1.4300 | 0.5080 | -2.8148 | 0.0049 | -2.4258 | -0.4343 |
| original_glcm_Correlation | -0.9976 | 0.5436 | -1.8351 | 0.0665 | -2.0631 | 0.0679 |
| original_glcm_Imc1 | -1.9296 | 0.7357 | -2.6229 | 0.0087 | -3.3715 | -0.4877 |
| original_glcm_Imc2 | -0.7959 | 0.5790 | -1.3746 | 0.1692 | -1.9307 | 0.3389 |
| original_glcm_MaximumProbability | 0.0279 | 0.3796 | 0.0736 | 0.9413 | -0.7161 | 0.7720 |
| original_gldm_LargeDependenceHighGrayLevelEmphasis | 1.4160 | 0.7543 | 1.8773 | 0.0605 | -0.0624 | 2.8943 |
| original_glrlm_LongRunHighGrayLevelEmphasis | -0.1794 | 0.7819 | -0.2294 | 0.8185 | -1.7119 | 1.3531 |
| original_glrlm_RunVariance | -2.1700 | 0.5892 | -3.6830 | 0.0002 | -3.3248 | -1.0152 |
| original_glszm_GrayLevelNonUniformity | 0.3895 | 0.3913 | 0.9953 | 0.3196 | -0.3774 | 1.1564 |
| original_glszm_HighGrayLevelZoneEmphasis | 0.1440 | 0.4866 | 0.2958 | 0.7674 | -0.8097 | 1.0977 |
| original_glszm_LargeAreaLowGrayLevelEmphasis | -0.0198 | 0.3422 | -0.0578 | 0.9539 | -0.6904 | 0.6509 |
| original_glszm_SmallAreaLowGrayLevelEmphasis | 0.0908 | 0.4116 | 0.2206 | 0.8254 | -0.7158 | 0.8974 |
| original_glszm_ZoneEntropy | 0.1655 | 0.4196 | 0.3945 | 0.6932 | -0.6568 | 0.9879 |
| original_ngtdm_Complexity | 0.3056 | 0.7733 | 0.3952 | 0.6927 | -1.2100 | 1.8212 |
| original_shape_MeshVolume | -0.6321 | 0.4047 | -1.5619 | 0.1183 | -1.4252 | 0.1611 |

Table *c* (Row 1-20 radiomic characteristics of T2)

| variables | Coef. | Std.Err. | z | P>\|z\| | [0.025 | 0.975] |
| --- | --- | --- | --- | --- | --- | --- |
| intercept | -0.2991 | 0.3113 | -0.9609 | 0.3366 | -0.9093 | 0.3110 |
| original_firstorder_Maximum | 0.4816 | 0.9493 | 0.5073 | 0.6119 | -1.3791 | 2.3423 |
| original_firstorder_Range | 0.4106 | 0.5750 | 0.7141 | 0.4752 | -0.7164 | 1.5376 |
| original_firstorder_RobustMeanAbsoluteDeviation | -2.2210 | 0.9984 | -2.2246 | 0.0261 | -4.1778 | -0.2642 |
| original_firstorder_Uniformity | -0.3046 | 0.5273 | -0.5777 | 0.5634 | -1.3380 | 0.7288 |
| original_glcm_ClusterShade | -1.1056 | 0.4789 | -2.3087 | 0.0210 | -2.0442 | -0.1670 |
| original_glcm_DifferenceVariance | -0.0922 | 0.4322 | -0.2133 | 0.8311 | -0.9392 | 0.7549 |
| original_gldm_HighGrayLevelEmphasis | -0.0751 | 0.8767 | -0.0856 | 0.9318 | -1.7934 | 1.6432 |
| original_gldm_SmallDependenceLowGrayLevelEmphasis | 0.9326 | 0.5241 | 1.7795 | 0.0752 | -0.0946 | 1.9599 |
| original_glrlm_RunLengthNonUniformityNormalized | -0.9298 | 0.8933 | -1.0408 | 0.2980 | -2.6807 | 0.8211 |
| original_glszm_LargeAreaHighGrayLevelEmphasis | 0.0772 | 0.5180 | 0.1491 | 0.8815 | -0.9381 | 1.0925 |
| original_ngtdm_Busyness | 1.6283 | 0.7688 | 2.1179 | 0.0342 | 0.1214 | 3.1351 |
| original_ngtdm_Coarseness | -0.1501 | 0.5582 | -0.2688 | 0.7881 | -1.2441 | 0.9440 |
| original_ngtdm_Complexity | -0.0768 | 1.0753 | -0.0714 | 0.9431 | -2.1844 | 2.0309 |
| original_ngtdm_Strength | -0.7926 | 0.5120 | -1.5480 | 0.1216 | -1.7961 | 0.2109 |
| original_shape_Elongation | 0.2537 | 0.5743 | 0.4418 | 0.6587 | -0.8719 | 1.3793 |
| original_shape_MajorAxisLength | 0.8382 | 0.6207 | 1.3505 | 0.1769 | -0.3783 | 2.0548 |
| original_shape_Maximum2DDiameterColumn | -0.0114 | 0.5673 | -0.0200 | 0.9840 | -1.1232 | 1.1005 |
| original_shape_Maximum2DDiameterRow | 0.5015 | 0.6346 | 0.7902 | 0.4294 | -0.7423 | 1.7453 |
| original_shape_Maximum3DDiameter | -0.7544 | 0.9757 | -0.7732 | 0.4394 | -2.6668 | 1.1579 |
| original_shape_VoxelVolume | -0.3811 | 0.4101 | -0.9294 | 0.3527 | -1.1848 | 0.4226 |
| original_firstorder_MeanAbsoluteDeviation | 1.7513 | 0.6892 | 2.5410 | 0.0111 | 0.4005 | 3.1022 |
| original_firstorder_Minimum | -0.6162 | 0.6215 | -0.9914 | 0.3215 | -1.8343 | 0.6019 |
| original_glcm_ClusterShade | 1.7369 | 0.6992 | 2.4839 | 0.0130 | 0.3664 | 3.1073 |
| original_glcm_DifferenceVariance | -1.2287 | 0.5817 | -2.1121 | 0.0347 | -2.3689 | -0.0885 |
| original_glcm_Idmn | -1.8897 | 0.7324 | -2.5800 | 0.0099 | -3.3252 | -0.4541 |
| original_glcm_JointEntropy | -1.4948 | 0.6196 | -2.4126 | 0.0158 | -2.7091 | -0.2804 |
| original_glcm_MCC | -2.8914 | 1.0883 | -2.6569 | 0.0079 | -5.0243 | -0.7584 |
| original_gldm_LargeDependenceLowGrayLevelEmphasis | 1.6765 | 1.2034 | 1.3932 | 0.1636 | -0.6821 | 4.0352 |
| original_glszm_GrayLevelNonUniformityNormalized | -3.3206 | 1.7196 | -1.9310 | 0.0535 | -6.6910 | 0.0499 |
| original_glszm_LargeAreaLowGrayLevelEmphasis | 0.2913 | 0.5237 | 0.5563 | 0.5780 | -0.7351 | 1.3178 |
| original_glszm_ZoneEntropy | 0.5912 | 0.5221 | 1.1324 | 0.2575 | -0.4320 | 1.6145 |
| original_glszm_ZoneVariance | -0.9115 | 0.5021 | -1.8153 | 0.0695 | -1.8956 | 0.0726 |
| original_shape_Elongation | 0.4796 | 0.6397 | 0.7497 | 0.4534 | -0.7741 | 1.7333 |
| original_shape_MajorAxisLength | 0.6458 | 0.5513 | 1.1714 | 0.2415 | -0.4348 | 1.7263 |

**Supplementary Material 4.** Radscore formula of each model

*T2(Radscore)*= -0.1830+（-0.3624×original_firstorder_Energy）+（0.1195×original_firstorder_Minimum）+（-0.1336×original_firstorder_Skewness）+（-0.1393×original_glcm_Contrast）+（-0.6420×original_glcm_Correlation）+（-1.0417×original_glcm_Imc1）+（-0.0048×original_glcm_MaximumProbability）+（1.8214×original_gldm_DependenceNonUniformityNormalized）+（1.2162×original_gldm_LargeDependenceHighGrayLevelEmphasis）+（-0.0083×original_glrlm_LongRunLowGrayLevelEmphasis）+（1.0921×original_glszm_GrayLevelNonUniformity）+（0.0283×original_glszm_LargeAreaHighGrayLevelEmphasis）+（0.0414×original_glszm_LowGrayLevelZoneEmphasis）+（-1.4669×original_glszm_SmallAreaEmphasis）+（-0.1827×original_glszm_SmallAreaLowGrayLevelEmphasis）+（-1.3275×original_glszm_ZoneEntropy）+（1.5798×original_ngtdm_Strength）+（0.2971×original_shape_Elongation）+（0.5073×original_shape_Flatness）+（0.1340×original_shape_Maximum2DDiameterColumn）+（-0.5611×original_shape_Sphericity）

*CET1(Radscore)*= -0.0857+（-1.9846×original_firstorder_Maximum）+（0.5740×original_firstorder_Minimum）+（2.0524×original_firstorder_Skewness）+（0.4558×original_firstorder_TotalEnergy）+（-1.4300×original_glcm_ClusterShade）+（-0.9976×original_glcm_Correlation）+（-1.9296×original_glcm_Imc1）+（-0.7959×original_glcm_Imc2）+（-0.7959×original_glcm_Imc2）+（0.0279×original_glcm_MaximumProb）+（1.4160×original_gldm_LargeDependenceHighGrayLevelEmphasis）+（-0.1794×original_glrlm_LongRunHighGrayLevelEmphasis）+（-2.1700×original_glrlm_RunVariance）+（0.3895×original_glszm_GrayLevelNonUniformity）+（0.1440×original_glszm_HighGrayLevelZoneEmphasis）+（-0.0198 ×original_glszm_LargeAreaLowGrayLevelEmphasis）+（0.0908×original_glszm_SmallAreaLowGrayLevelEmphasis）+（0.1655×original_glszm_ZoneEntropy）+（0.3056×original_ngtdm_Complexity）+（-0.6321×original_shape_MeshVolume）

*Combing(Radscore)*= -0.2991+（0.4816×original_firstorder_Maximum）+（0.4106×original_firstorder_Range）+（-2.2210×original_firstorder_RobustMeanAbsoluteDeviation）+（-0.3046×original_firstorder_Uniformity）+（-1.1056×original_glcm_ClusterShade）+（-0.0922×original_glcm_DifferenceVariance）+（-0.0751×original_gldm_HighGrayLevelEmphasis）+（0.9326×original_gldm_SmallDependenceLowGrayLevelEmphasis）+（-0.9298×original_glrlm_RunLengthNonUniformityNormalized）+（0.0772×original_glszm_LargeAreaHighGrayLevelEmphasis）+（1.6283×original_ngtdm_Busyness）+（-0.1501×original_ngtdm_Coarseness）+（-0.0768×original_ngtdm_Complexity）+（-0.7926×original_ngtdm_Strength）+（0.2537×original_shape_Elongation）+（0.8382×original_shape_MajorAxisLength）+（-0.0114×original_shape_Maximum2DDiameterColumn）+（0.5015×original_shape_Maximum2DDiameterRow）+（-0.7544×original_shape_Maximum3DDiameter）+（-0.3811×original_shape_VoxelVolume）+（1.7513×original_firstorder_MeanAbsoluteDeviation）+（-0.6162×original_firstorder_Minimum）+（1.7369×original_glcm_ClusterShade）+（-1.2287×original_glcm_DifferenceVariance）+（-1.8897×original_glcm_Idmn）+（-1.4948×original_glcm_JointEntropy）+（-2.8914×original_glcm_MCC）+（1.6765×original_gldm_LargeDependenceLowGrayLevelEmphasis）+（-3.3206×original_glszm_GrayLevelNonUniformityNormalized）+（0.2913×original_glszm_LargeAreaLowGrayLevelEmphasis）+（0.5912×original_glszm_ZoneEntropy）+（-0.9115×original_glszm_ZoneVariance）+（0.4796×original_shape_Elongation）+（0.6458×original_shape_MajorAxisLength）

**Supplementary Material 5.** *a* and *b* respectively is the scores of the radiological features of the T2 image in the training group and the validation group. *c* and *d* respectively is the scores of the radiological features of the CET1 image in the training group and the validation group. *e* and *f* respectively is the scores of the radiological features of the combine image in the training group and the validation group.


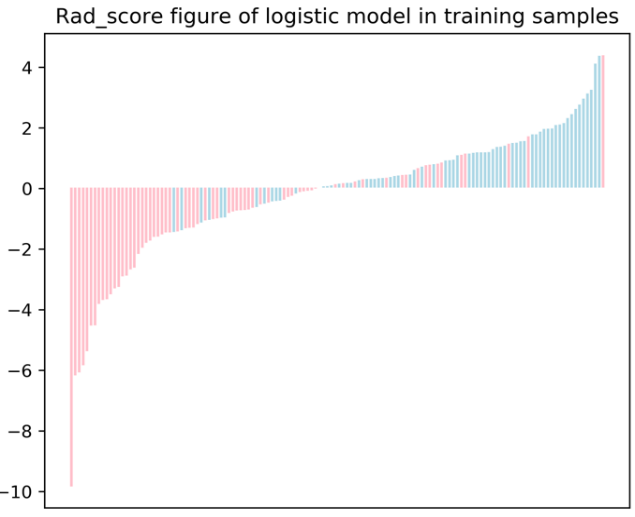

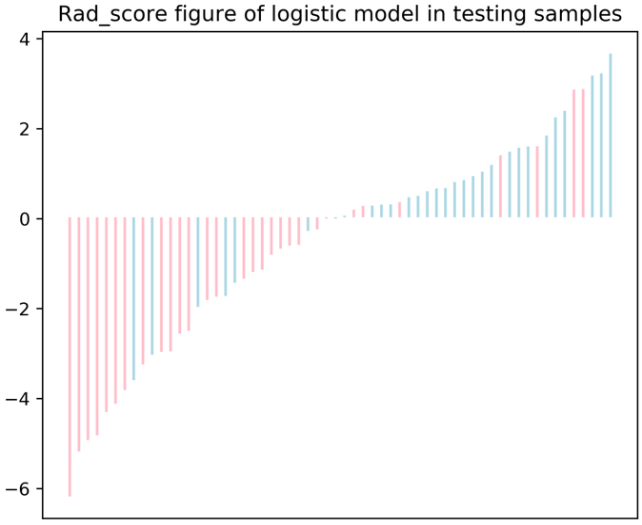


*a b*


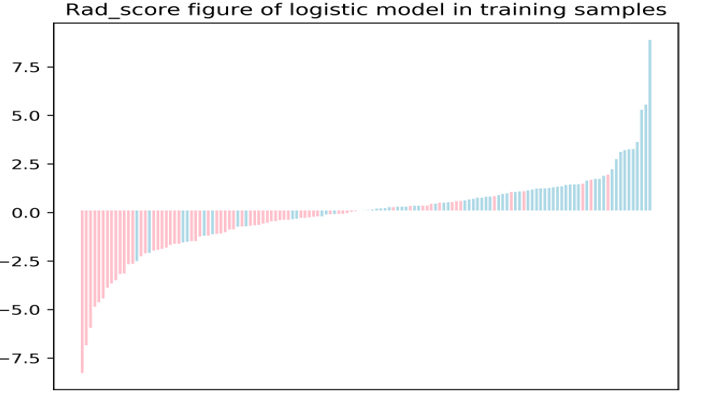

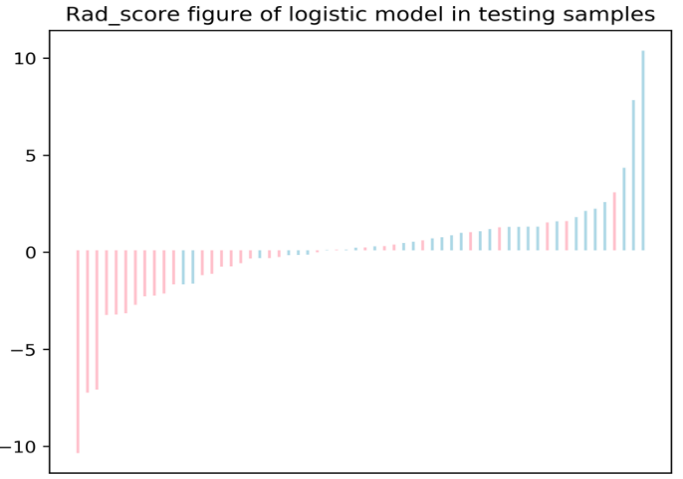


*c d*


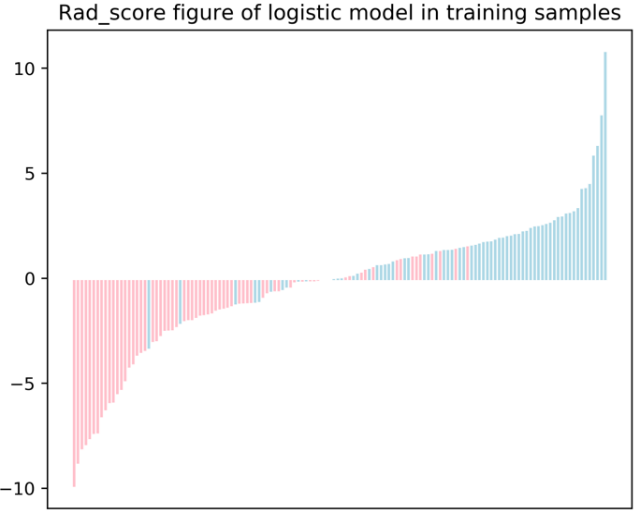

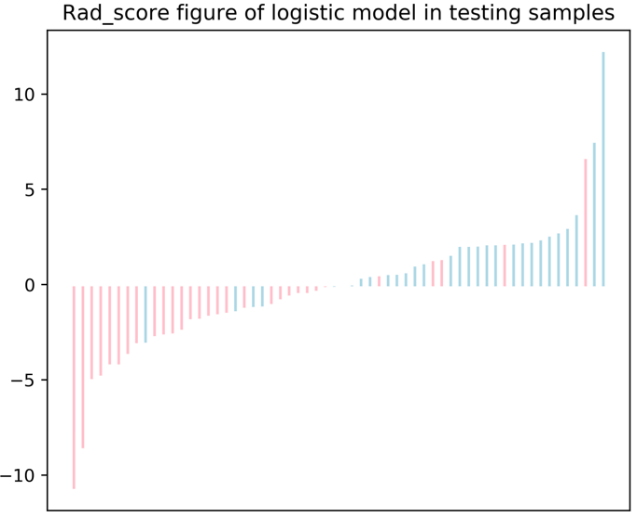


*e f*

**Supplementary Material 6.** Masson staining results of pathological sections of pituitary macroadenoma (*a* is masson staining results of pathological sections of hard patients, and the staining area accounts for 46.1%. *b* is masson staining results of pathological sections of soft patients, and the staining area accounts for 11.95%)


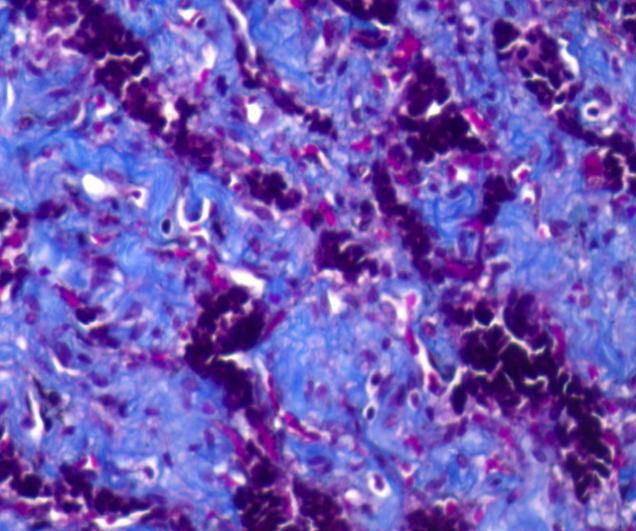

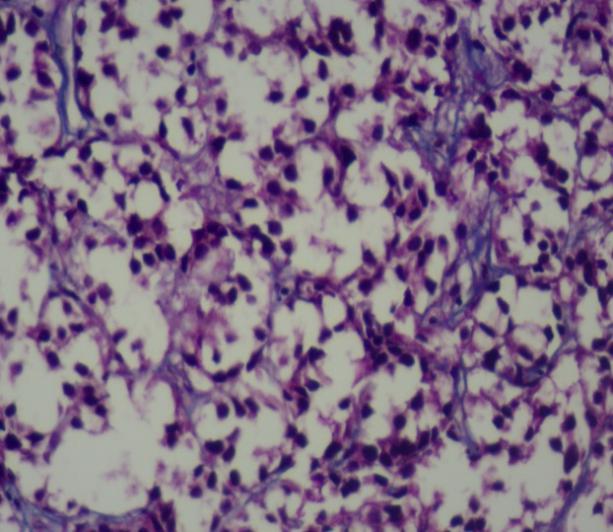


*a b*
