# Supplementary material for: MR radiomics in assessment of consistency of pituitary macroadenoma: can T1-weighted contrast enhanced image improve diagnostic performance of T2-weighted image?
Source: Front Oncol. 2025 Sep 3;15:1539432. doi: 10.3389/fonc.2025.1539432 (PMC12440780; doi:10.3389/fonc.2025.1539432)
Supplement: Supplementary file 2 [file DataSheet2.docx]

install.packages("verification")

install.packages("pROC")

install.packages("rms")

install.packages("glmnet")

library(verification)

library(pROC) # ROC

library(rms) # nomogram

library(glmnet) # cv.glmnet

#---------------------------------- ROC -----------------------------------------

rm(list=ls()) # 清空

setwd("C:/Users/212481425/Desktop/zoumenghong/0806_zoumenghong")

getwd()

df_1<- read.csv("CET1_Seed_60_0.70.csv",header = T)

df_2<- read.csv("T2_Seed_18_0.70.csv",header = T)

df_com<- read.csv("combine_seed_123.csv",header = T)

roc_1_train<-roc(df_1$label[which(df_1[,2]== 1) ],df_1$rad_score[which(df_1[,2]== 1) ],ci=T)

roc_1_test<-roc(df_1$label[which(df_1[,2]== 0) ],df_1$rad_score[which(df_1[,2]== 0) ],ci=T)

roc_2_train<-roc(df_2$label[which(df_2[,2]== 1) ],df_2$rad_score[which(df_2[,2]== 1) ],ci=T)

roc_2_test<-roc(df_2$label[which(df_2[,2]== 0) ],df_2$rad_score[which(df_2[,2]== 0) ],ci=T)

roc_com_train<-roc(df_com$label[which(df_com[,2]== 1) ],df_com$rad_score[which(df_com[,2]== 1) ],ci=T)

roc_com_test<-roc(df_com$label[which(df_com[,2]== 0) ],df_com$rad_score[which(df_com[,2]== 0) ],ci=T)

#----------- delong test ----------------------

roc.test(roc_1_test,roc_1_train)

roc.test(roc_2_test,roc_2_train)

roc.test(roc_com_test,roc_com_train)

roc.test(roc_1_test,roc_2_test)

roc.test(roc_1_test,roc_com_test)

roc.test(roc_2_test,roc_com_test)

roc.test(roc_1_train,roc_2_train)

roc.test(roc_1_train,roc_com_train)

roc.test(roc_2_train,roc_com_train)

#----------- PLOT Roc ---------------------------

tiff(file = "C:/Users/212481425/Desktop/ROC_train_600.tiff", res = 600,width =4500, height = 4500)

plot(roc_1_train, col=1, main="The ROC curves in training dataset")

plot(roc_2_train, col=4,add=T)

plot(roc_com_train,col=2,add=T)

legend(0.75,0.2,

c("CET1: AUC=0.828,95% CI=0.758-0.897",

"T2: AUC=0.845,95% CI=0.781-0.910",

"Combine: AUC=0.902,95% CI=0.853-0.950"

),

border=3,

cex=1.1,

text.width = 0.65,

col=c(1,4,2),

lty= 1,

lwd= 3)

dev.off()

tiff(file = "C:/Users/212481425/Desktop/ROC_test_600.tiff", res = 600,width =4500, height = 4500)

plot(roc_1_test, col=1, main="The ROC curves in test dataset")

plot(roc_2_test, col=4,add=T)

plot(roc_com_test,col=2,add=T)

legend(0.75,0.2,

c("CET1: AUC=0.802,95% CI=0.690-0.915",

"T2: AUC=0.791,95% CI=0.671-0.911",

"Combine: AUC=0.862,95% CI=0.765-0.960"

),

border=3,

cex=1.1,

text.width = 0.65,

col=c(1,4,2),

lty= 1,

lwd= 3)

dev.off()

#------------------------------------ DCA ----------------------------------

library(rmda)

rm(list=ls()) # 清空

setwd("C:/Users/212481425/Desktop/liutong/0205_liutong_radiomics_analysis/12_added_for_dca")

getwd()

df_train<- read.csv("for_combine_train.csv",header = T)

df_test<- read.csv("for_combine_test.csv",header = T)

names(df_1)

df_1[which(df_1[,2]== 1), c(1,3)]

dca_1_train <- decision_curve(label~rad_score,data=df_1[which(df_1[,2]== 1), c(1,3)])

dca_2_train <- decision_curve(label~rad_score,data=df_2[which(df_2[,2]== 1), c(1,3)])

dca_com_train <- decision_curve(label~rad_score,data=df_com[which(df_com[,2]== 1), c(1,3)])

dca_1_test <- decision_curve(label~rad_score,data=df_1[which(df_1[,2]== 0), c(1,3)])

dca_2_test<- decision_curve(label~rad_score,data=df_2[which(df_2[,2]== 0), c(1,3)])

dca_com_test <- decision_curve(label~rad_score,data=df_com[which(df_com[,2]== 0), c(1,3)])

tiff(filename = "C:/Users/212481425/Desktop/DCA_train_600dpi.tiff",width=5000,height = 3500,res=600)

plot_decision_curve(list( dca_1_train, dca_2_train,dca_com_train),

confidence.intervals = F,

col = c(1,4,2),

curve.names = c('CET1','T1','Combine'),

lty=c(1,1,1),

lwd=3,

xlab="Threshold Probability",

ylab="Net Benefit",

legend.position = 'topright',

cost.benefits = T

)

dev.off()

tiff(filename = "C:/Users/212481425/Desktop/DCA_test_600dpi.tiff",width=5000,height = 3500,res=600)

plot_decision_curve(list( dca_1_test, dca_2_test,dca_com_test),

confidence.intervals = F,

col = c(1,4,2),

curve.names = c('CET1','T1','Combine'),

lty=c(1,1,1),

lwd=3,

xlab="Threshold Probability",

ylab="Net Benefit",

legend.position = 'topright',

cost.benefits = T

)

dev.off()

#------------------------------- calibration ------------------------------------

library(ModelGood)

library(DescTools)

flrm_1_train<-lrm(label~.,data=df_1[which(df_1[,2]== 1), c(1,3)],x=T,y=T )

flrm_1_test<-lrm(label~.,data=df_1[which(df_1[,2]== 0), c(1,3)],x=T,y=T )

flrm_2_train<-lrm(label~.,data=df_2[which(df_2[,2]== 1), c(1,3)],x=T,y=T )

flrm_2_test<-lrm(label~.,data=df_2[which(df_2[,2]== 0), c(1,3)],x=T,y=T )

flrm_com_train<-lrm(label~.,data=df_com[which(df_com[,2]== 1), c(1,3)],x=T,y=T )

flrm_com_test<-lrm(label~.,data=df_com[which(df_com[,2]== 0), c(1,3)],x=T,y=T )

tiff(filename = "C:/Users/212481425/Desktop/Calibration_train_600dpi.tiff",width=4500,height = 4500,res=600)

cal_train_1<-calPlot2( flrm_1_train,

col=1,

xlab="Predicted Probability",

ylab="Actual Probability",

showY = F,

lty=1,

legend = F)

cal_train_2<-calPlot2( flrm_2_train,

col=c(4),

add=T,

showY = F,

lty=1,

legend = F)

cal_train_com<-calPlot2( flrm_com_train,

col=2,

add=T,

showY = F,

lty=1,

legend = F)

HosmerLemeshowTest(cal_train_1$Frame$lrm,cal_train_1$Frame$jack)

HosmerLemeshowTest(cal_train_2$Frame$lrm,cal_train_2$Frame$jack)

HosmerLemeshowTest(cal_train_com$Frame$lrm,cal_train_com$Frame$jack)

legend(0.35,0.2,

c("CET1: H-L test P value=0.478",

"T2: H-L test P value=0.644 ",

"Combine: H-L test P value=0.328"

),

border=3,

cex=1.1,

text.width = 0.5,

col=c(1,4,2),

lty= 1,

lwd= 3)

dev.off()

tiff(filename = "C:/Users/212481425/Desktop/Calibration_test_600dpi.tiff",width=4500,height = 4500,res=600)

cal_test_1<-calPlot2( flrm_1_test,

col=1,

xlab="Predicted Probability",

ylab="Actual Probability",

showY = F,

lty=1,

legend = F)

cal_test_2<-calPlot2( flrm_2_test,

col=c(4),

add=T,

showY = F,

lty=1,

legend = F)

cal_test_com<-calPlot2( flrm_com_test,

col=2,

add=T,

showY = F,

lty=1,

legend = F)

HosmerLemeshowTest(cal_test_1$Frame$lrm,cal_test_1$Frame$jack)

HosmerLemeshowTest(cal_test_2$Frame$lrm,cal_test_2$Frame$jack)

HosmerLemeshowTest(cal_test_com$Frame$lrm,cal_test_com$Frame$jack)

legend(0.35,0.2,

c("CET1: H-L test P value=0.918",

"T2: H-L test P value=0.451 ",

"Combine: H-L test P value=0.272"

),

border=3,

cex=1.1,

text.width = 0.5,

col=c(1,4,2),

lty= 1,

lwd= 3)

dev.off()

#------------- Stratify ---------------------------------

rm(list=ls()) # 清空

setwd("C:/Users/212481425/Desktop/zoumenghong/0513_zoumenghong")

getwd()

df_1<- read.csv("for_stratify_field_strength_1.5.csv",header = T)

df_2<- read.csv("for_stratify_field_strength_3.0.csv",header = T)

df_g<- read.csv("for_stratify_vendor_G.csv",header = T)

df_p<- read.csv("for_stratify_vendor_P.csv",header = T)

df_s<- read.csv("for_stratify_vendor_S.csv",header = T)

roc_1_1<-roc(df_1$label, df_1$CET1_Score,ci=T)

roc_1_2<-roc(df_1$label, df_1$T2_Score,ci=T)

roc_1_3<-roc(df_1$label, df_1$Combine_Score,ci=T)

roc_2_1<-roc(df_2$label, df_2$CET1_Score,ci=T)

roc_2_2<-roc(df_2$label, df_2$T2_Score,ci=T)

roc_2_3<-roc(df_2$label, df_2$Combine_Score,ci=T)

roc_g_1<-roc(df_g$label, df_g$CET1_Score,ci=T)

roc_g_2<-roc(df_g$label, df_g$T2_Score,ci=T)

roc_g_3<-roc(df_g$label, df_g$Combine_Score,ci=T)

roc_p_1<-roc(df_p$label, df_p$CET1_Score,ci=T)

roc_p_2<-roc(df_p$label, df_p$T2_Score,ci=T)

roc_p_3<-roc(df_p$label, df_p$Combine_Score,ci=T)

roc_s_1<-roc(df_s$label, df_s$CET1_Score,ci=T)

roc_s_2<-roc(df_s$label, df_s$T2_Score,ci=T)

roc_s_3<-roc(df_s$label, df_s$Combine_Score,ci=T)

#--------------------------------------------------------------------------------------------

roc.test(roc_1_1,roc_2_1)

roc.test(roc_1_2,roc_2_2)

roc.test(roc_1_3,roc_2_3)

roc.test(roc_g_1,roc_p_1)

roc.test(roc_p_1,roc_s_1)

roc.test(roc_g_1,roc_s_1)

roc.test(roc_g_2,roc_p_2)

roc.test(roc_p_2,roc_s_2)

roc.test(roc_g_2,roc_s_2)

roc.test(roc_g_3,roc_p_3)

roc.test(roc_p_3,roc_s_3)

roc.test(roc_g_3,roc_s_3)

#------------------------------ plot new ---------------------------------

tiff(file = "C:/Users/212481425/Desktop/ROC_strength_600.tiff", res = 1200,width =9000, height = 9000)

plot(roc_1_1, col=1, main="The ROC curves for different field strength")

plot(roc_2_1, col=8,add=T)

plot(roc_1_2, col=3, add=T)

plot(roc_2_2, col=4,add=T)

plot(roc_1_3, col=6, add=T)

plot(roc_2_3, col=7,add=T)

legend(0.75,0.25,

c("CET1-1.5T: AUC=0.500,95% CI=0.226-0.774",

"CET1-3.0T: AUC=0.610,95% CI=0.498-0.722",

"T2-1.5T: AUC=0.763,95% CI=0.522-1.000",

"T2-3.0T: AUC=0.834,95% CI=0.749-0.919",

"Combine-1.5T: AUC=0.581,95% CI=0.289-0.873",

"Combine-3.0T: AUC=0.563,95% CI=0.433-0.693"

),

border=3,

cex=0.95,

text.width = 0.65,

col=c(1,8,3,4,6,7),

lty= 1,

lwd= 3)

dev.off()

tiff(file = "C:/Users/212481425/Desktop/ROC_vendor_600.tiff", res = 1200,width =9000, height = 9000)

plot(roc_p_1, col=1, main="The ROC curves for different vendor")

plot(roc_s_1, col=2,add=T)

plot(roc_p_2, col=3,add=T)

plot(roc_s_2, col=4,add=T)

plot(roc_p_3, col=7,add=T)

plot(roc_s_3, col=6,add=T)

legend(0.75,0.25,

c(

"CET1-Philips: AUC=0.541,95% CI=0.361-0.721",

"CET1-Siemens: AUC=0.608,95% CI=0.456-0.730",

"T2-Philips: AUC=0.841,95% CI=0.703-0.978",

"T2-Siemens: AUC=0.832,95% CI=0.741-0.922",

"Combine-Philips: AUC=0.527,95% CI=0.335-0.719",

"Combine-Siemens: AUC=0.532,95% CI=0.375-0.688"

),

border=3,

cex=0.95,

text.width = 0.65,

col=c(1,2,3,4,7,6),

lty= 1,

lwd= 3)

dev.off()

#--------------------------------------------------------------------------------------------

tiff(file = "C:/Users/212481425/Desktop/ROC_1_600.tiff", res = 1200,width =9000, height = 9000)

plot(roc_1_1, col=1, main="The ROC curves of CET1 Score")

plot(roc_2_1, col=4,add=T)

legend(0.75,0.2,

c("1.5T: AUC=0.500,95% CI=0.226-0.774",

"3.0T: AUC=0.610,95% CI=0.498-0.722"

),

border=3,

cex=1.1,

text.width = 0.65,

col=c(1,4),

lty= 1,

lwd= 3)

dev.off()

tiff(file = "C:/Users/212481425/Desktop/ROC_2_600.tiff", res = 1200,width =9000, height = 9000)

plot(roc_1_2, col=1, main="The ROC curves of T2 Score")

plot(roc_2_2, col=4,add=T)

legend(0.75,0.2,

c("1.5T: AUC=0.763,95% CI=0.522-1.000",

"3.0T: AUC=0.834,95% CI=0.749-0.919"

),

border=3,

cex=1.1,

text.width = 0.65,

col=c(1,4),

lty= 1,

lwd= 3)

dev.off()

tiff(file = "C:/Users/212481425/Desktop/ROC_3_600.tiff", res = 1200,width =9000, height = 9000)

plot(roc_1_3, col=1, main="The ROC curves of Combine Score")

plot(roc_2_3, col=4,add=T)

legend(0.75,0.2,

c("1.5T: AUC=0.581,95% CI=0.289-0.873",

"3.0T: AUC=0.563,95% CI=0.433-0.693"

),

border=3,

cex=1.1,

text.width = 0.65,

col=c(1,4),

lty= 1,

lwd= 3)

dev.off()

#----------------------

tiff(file = "C:/Users/212481425/Desktop/ROC_v1_600.tiff", res = 1200,width =9000, height = 9000)

plot(roc_g_1, col=1, main="The ROC curves of CET1 Score")

plot(roc_p_1, col=4,add=T)

plot(roc_s_1, col=3,add=T)

legend(0.75,0.2,

c("GE: AUC=0.500,95% CI=0.000-1.000",

"Philips: AUC=0.541,95% CI=0.361-0.721",

"Siemens: AUC=0.608,95% CI=0.456-0.730"

),

border=3,

cex=1.1,

text.width = 0.65,

col=c(1,4,3),

lty= 1,

lwd= 3)

dev.off()

tiff(file = "C:/Users/212481425/Desktop/ROC_v2_600.tiff", res = 1200,width =9000, height = 9000)

plot(roc_g_2, col=1, main="The ROC curves of T2 Score")

plot(roc_p_2, col=4,add=T)

plot(roc_s_2, col=3,add=T)

legend(0.75,0.2,

c("GE: AUC=0.500,95% CI=0.000-1.000",

"Philips: AUC=0.841,95% CI=0.703-0.978",

"Siemens: AUC=0.832,95% CI=0.741-0.922"

),

border=3,

cex=1.1,

text.width = 0.65,

col=c(1,4,3),

lty= 1,

lwd= 3)

dev.off()

tiff(file = "C:/Users/212481425/Desktop/ROC_v3_600.tiff", res = 1200,width =9000, height = 9000)

plot(roc_g_3, col=1, main="The ROC curves of Combine Score")

plot(roc_p_3, col=4,add=T)

plot(roc_s_3, col=3,add=T)

legend(0.75,0.2,

c("GE: AUC=0.833,95% CI=0.371-1.000",

"Philips: AUC=0.527,95% CI=0.335-0.719",

"Siemens: AUC=0.532,95% CI=0.375-0.688"

),

border=3,

cex=1.1,

text.width = 0.65,

col=c(1,4,3),

lty= 1,

lwd= 3)

dev.off()

#--------------------------------------

tiff(file = "C:/Users/212481425/Desktop/ROC_v11_600.tiff", res = 1200,width =9000, height = 9000)

plot(roc_s_1, col=1, main="The ROC curves of CET1 Score")

plot(roc_p_1, col=4,add=T)

legend(0.75,0.2,

c("Siemens: AUC=0.608,95% CI=0.456-0.730",

"Philips: AUC=0.541,95% CI=0.361-0.721"

),

border=3,

cex=1.1,

text.width = 0.65,

col=c(1,4),

lty= 1,

lwd= 3)

dev.off()

tiff(file = "C:/Users/212481425/Desktop/ROC_v22_600.tiff", res = 1200,width =9000, height = 9000)

plot(roc_s_2, col=1, main="The ROC curves of T2 Score")

plot(roc_p_2, col=4,add=T)

legend(0.75,0.2,

c("Siemens: AUC=0.832,95% CI=0.741-0.922",

"Philips: AUC=0.841,95% CI=0.703-0.978"

),

border=3,

cex=1.1,

text.width = 0.65,

col=c(1,4),

lty= 1,

lwd= 3)

dev.off()

tiff(file = "C:/Users/212481425/Desktop/ROC_v33_600.tiff", res = 1200,width =9000, height = 9000)

plot(roc_s_3, col=1, main="The ROC curves of Combine Score")

plot(roc_p_3, col=4,add=T)

legend(0.75,0.2,

c("Siemens: AUC=0.532,95% CI=0.375-0.688",

"Philips: AUC=0.527,95% CI=0.335-0.719"

),

border=3,

cex=1.1,

text.width = 0.65,

col=c(1,4),

lty= 1,

lwd= 3)

dev.off()

.

#--------------------- Cutoff -------------------------------------

plot(roc_1_train,print.thres=T)

plot(roc_2_train,print.thres=T)

plot(roc_3_train,print.thres=T)

plot(roc_4_train,print.thres=T)

plot(roc_5_train,print.thres=T)

plot(roc_6_train,print.thres=T)

plot(roc_7_train,print.thres=T)

plot(roc_8_train,print.thres=T)

plot(roc_9_train,print.thres=T)

plot(roc_10_train,print.thres=T)

#---------------------------------- vio plot ----------------------

names(df_1)

library(ggplot2)

library(ggpubr)

tiff(filename = "C:/Users/212481425/Desktop/1_train_600dpi.tiff",width=3500,height = 3500,res=600)

ggboxplot(x = "label",

y = "rad_score",

data=df_1[which(df_1[,2]==1),],

add = 'jitter',

color = 'label',

palette = "default") +

ylim(-4, 6) +

stat_compare_means(method = 'wilcox.test') +

geom_hline(yintercept = 0.039) + theme_bw()

dev.off()

tiff(filename = "C:/Users/212481425/Desktop/1_test_600dpi.tiff",width=3500,height = 3500,res=600)

ggboxplot(x = "label",

y = "rad_score",

data=df_1[which(df_1[,2]==0),],

add = 'jitter',

color = 'label',

palette = "default") +

ylim(-4, 6) +

stat_compare_means(method = 'wilcox.test') +

geom_hline(yintercept = 0.039) + theme_bw()

dev.off()

#------------- 2

tiff(filename = "C:/Users/212481425/Desktop/2_train_600dpi.tiff",width=3500,height = 3500,res=600)

ggboxplot(x = "label",

y = "rad_score",

data=df_2[which(df_2[,2]==1),],

add = 'jitter',

color = 'label',

palette = "default") +

ylim(-9, 5) +

stat_compare_means(method = 'wilcox.test') +

geom_hline(yintercept = 0.601) + theme_bw()

dev.off()

tiff(filename = "C:/Users/212481425/Desktop/2_test_600dpi.tiff",width=3500,height = 3500,res=600)

ggboxplot(x = "label",

y = "rad_score",

data=df_2[which(df_2[,2]==0),],

add = 'jitter',

color = 'label',

palette = "default") +

ylim(-9, 5) +

stat_compare_means(method = 'wilcox.test') +

geom_hline(yintercept = 0.601) + theme_bw()

dev.off()

#---------------------- 3

tiff(filename = "C:/Users/212481425/Desktop/3_train_600dpi.tiff",width=3500,height = 3500,res=600)

ggboxplot(x = "label",

y = "rad_score",

data=df_3[which(df_3[,2]==1),],

add = 'jitter',

color = 'label',

palette = "default") +

ylim(-10, 5) +

stat_compare_means(method = 'wilcox.test') +

geom_hline(yintercept = -0.645) + theme_bw()

dev.off()

tiff(filename = "C:/Users/212481425/Desktop/3_test_600dpi.tiff",width=3500,height = 3500,res=600)

ggboxplot(x = "label",

y = "rad_score",

data=df_3[which(df_3[,2]==0),],

add = 'jitter',

color = 'label',

palette = "default") +

ylim(-10, 5) +

stat_compare_means(method = 'wilcox.test') +

geom_hline(yintercept = -0.645) + theme_bw()

dev.off()

#------------------ 4

tiff(filename = "C:/Users/212481425/Desktop/4_train_600dpi.tiff",width=3500,height = 3500,res=600)

ggboxplot(x = "label",

y = "rad_score",

data=df_4[which(df_4[,2]==1),],

add = 'jitter',

color = 'label',

palette = "default") +

ylim(-15, 10) +

stat_compare_means(method = 'wilcox.test') +

geom_hline(yintercept = -0.466) + theme_bw()

dev.off()

tiff(filename = "C:/Users/212481425/Desktop/4_test_600dpi.tiff",width=3500,height = 3500,res=600)

ggboxplot(x = "label",

y = "rad_score",

data=df_4[which(df_4[,2]==0),],

add = 'jitter',

color = 'label',

palette = "default") +

ylim(-15, 10) +

stat_compare_means(method = 'wilcox.test') +

geom_hline(yintercept = -0.466) + theme_bw()

dev.off()

#-------------------- 5

tiff(filename = "C:/Users/212481425/Desktop/5_train_600dpi.tiff",width=3500,height = 3500,res=600)

ggboxplot(x = "label",

y = "rad_score",

data=df_5[which(df_5[,2]==1),],

add = 'jitter',

color = 'label',

palette = "default") +

ylim(-8, 3) +

stat_compare_means(method = 'wilcox.test') +

geom_hline(yintercept = -0.073) + theme_bw()

dev.off()

tiff(filename = "C:/Users/212481425/Desktop/5_test_600dpi.tiff",width=3500,height = 3500,res=600)

ggboxplot(x = "label",

y = "rad_score",

data=df_5[which(df_5[,2]==0),],

add = 'jitter',

color = 'label',

palette = "default") +

ylim(-8, 3) +

stat_compare_means(method = 'wilcox.test') +

geom_hline(yintercept = -0.073) + theme_bw()

dev.off()

#------------------------ 6

tiff(filename = "C:/Users/212481425/Desktop/6_train_600dpi.tiff",width=3500,height = 3500,res=600)

ggboxplot(x = "label",

y = "rad_score",

data=df_6[which(df_6[,2]==1),],

add = 'jitter',

color = 'label',

palette = "default") +

ylim(-5, 4) +

stat_compare_means(method = 'wilcox.test') +

geom_hline(yintercept = -0.297) + theme_bw()

dev.off()

tiff(filename = "C:/Users/212481425/Desktop/6_test_600dpi.tiff",width=3500,height = 3500,res=600)

ggboxplot(x = "label",

y = "rad_score",

data=df_6[which(df_6[,2]==0),],

add = 'jitter',

color = 'label',

palette = "default") +

ylim(-5, 4) +

stat_compare_means(method = 'wilcox.test') +

geom_hline(yintercept = -0.297) + theme_bw()

dev.off()

#----------------------------------- 7

tiff(filename = "C:/Users/212481425/Desktop/7_train_600dpi.tiff",width=3500,height = 3500,res=600)

ggboxplot(x = "label",

y = "rad_score",

data=df_7[which(df_7[,2]==1),],

add = 'jitter',

color = 'label',

palette = "default") +

ylim(-9, 5) +

stat_compare_means(method = 'wilcox.test') +

geom_hline(yintercept = -0.860) + theme_bw()

dev.off()

tiff(filename = "C:/Users/212481425/Desktop/7_test_600dpi.tiff",width=3500,height = 3500,res=600)

ggboxplot(x = "label",

y = "rad_score",

data=df_7[which(df_7[,2]==0),],

add = 'jitter',

color = 'label',

palette = "default") +

ylim(-9, 5) +

stat_compare_means(method = 'wilcox.test') +

geom_hline(yintercept = -0.860) + theme_bw()

dev.off()

#----------------------- 8

tiff(filename = "C:/Users/212481425/Desktop/8_train_600dpi.tiff",width=3500,height = 3500,res=600)

ggboxplot(x = "label",

y = "rad_score",

data=df_8[which(df_8[,2]==1),],

add = 'jitter',

color = 'label',

palette = "default") +

ylim(-9, 9) +

stat_compare_means(method = 'wilcox.test') +

geom_hline(yintercept = -0.206) + theme_bw()

dev.off()

tiff(filename = "C:/Users/212481425/Desktop/8_test_600dpi.tiff",width=3500,height = 3500,res=600)

ggboxplot(x = "label",

y = "rad_score",

data=df_8[which(df_8[,2]==0),],

add = 'jitter',

color = 'label',

palette = "default") +

ylim(-9, 9) +

stat_compare_means(method = 'wilcox.test') +

geom_hline(yintercept = -0.206) + theme_bw()

dev.off()

#------------------------------ 9

tiff(filename = "C:/Users/212481425/Desktop/9_train_600dpi.tiff",width=3500,height = 3500,res=600)

ggboxplot(x = "label",

y = "rad_score",

data=df_9[which(df_9[,2]==1),],

add = 'jitter',

color = 'label',

palette = "default") +

ylim(-6, 4) +

stat_compare_means(method = 'wilcox.test') +

geom_hline(yintercept = -0.538) + theme_bw()

dev.off()

tiff(filename = "C:/Users/212481425/Desktop/9_test_600dpi.tiff",width=3500,height = 3500,res=600)

ggboxplot(x = "label",

y = "rad_score",

data=df_9[which(df_9[,2]==0),],

add = 'jitter',

color = 'label',

palette = "default") +

ylim(-6, 4) +

stat_compare_means(method = 'wilcox.test') +

geom_hline(yintercept = -0.538) + theme_bw()

dev.off()

#-------------------------- 10

tiff(filename = "C:/Users/212481425/Desktop/10_train_600dpi.tiff",width=3500,height = 3500,res=600)

ggboxplot(x = "label",

y = "rad_score",

data=df_10[which(df_10[,2]==1),],

add = 'jitter',

color = 'label',

palette = "default") +

ylim(-8, 5) +

stat_compare_means(method = 'wilcox.test') +

geom_hline(yintercept = -0.355) + theme_bw()

dev.off()

tiff(filename = "C:/Users/212481425/Desktop/10_test_600dpi.tiff",width=3500,height = 3500,res=600)

ggboxplot(x = "label",

y = "rad_score",

data=df_10[which(df_10[,2]==0),],

add = 'jitter',

color = 'label',

palette = "default") +

ylim(-8, 5) +

stat_compare_means(method = 'wilcox.test') +

geom_hline(yintercept = -0.355) + theme_bw()

dev.off()

#-------------------------- combine box plot ------------

library(ggplot2)

library(ggpubr)

tiff(filename = "C:/Users/212481425/Desktop/combine_train_600dpi.tiff",width=3500,height = 3500,res=600)

ggboxplot(x = "label",

y = "Combine",

data=df_train,

add = 'jitter',

color = 'label',

palette = "default") +

ylim(-15, 10) +

stat_compare_means(method = 'wilcox.test') +

geom_hline(yintercept = -0.235) + theme_bw()

dev.off()

tiff(filename = "C:/Users/212481425/Desktop/combine_test_600dpi.tiff",width=3500,height = 3500,res=600)

ggboxplot(x = "label",

y = "Combine",

data=df_test,

add = 'jitter',

color = 'label',

palette = "default") +

ylim(-15, 10) +

stat_compare_means(method = 'wilcox.test') +

geom_hline(yintercept = -0.235) + theme_bw()

dev.off()

#------------------------------

setwd("C:/Users/212481425/Desktop")

getwd()

df<- read.csv("Combine DWI_conventional.csv",header = T)

roc_ADC<-roc(df$label,df$ADC,ci=T)

roc_D<-roc(df$label,df$D,ci=T)

roc_f<-roc(df$label,df$f,ci=T)

roc_D_star<-roc(df$label,df$D.,ci=T)

plot(roc_f,print.thres=T)

#------------------------ nomogram ------------

setwd("C:/Users/212481425/Desktop")

getwd()

df_train<- read.csv("for_nomogram.csv",header = T)

response_train<-as.factor(df_train$label)

train<-df_train[-1]

tiff(filename = "C:/Users/212481425/Desktop/nomogram_600dpi.tiff",width=5000,height = 2500,res=600)

# nomogram_train

{

dt <- data.frame(train)

ddist <- datadist(dt)

options(datadist="ddist")

flrm <- lrm(response_train ~ .,data=dt,x=T,y=T)

# va <- validate(flrm,method="boot",B=150,dxy=T,pr=T)

# cal <- calibrate(flrm,method="boot",B=150)

nomogplot <- nomogram(flrm,

fun=plogis,

fun.at=c(.0,.1, .5,.9),

lp=F,

funlabel="Probability")

par(mfrow=c(1,1))

plot(nomogplot)

}

dev.off()
