# Supplementary material for: MR radiomics in assessment of consistency of pituitary macroadenoma: can T1-weighted contrast enhanced image improve diagnostic performance of T2-weighted image?
Source: Front Oncol. 2025 Sep 3;15:1539432. doi: 10.3389/fonc.2025.1539432 (PMC12440780; doi:10.3389/fonc.2025.1539432)
Supplement: Supplementary file 3 [file DataSheet3.docx]

install.packages("verification")

install.packages("pROC")

install.packages("rms")

install.packages("glmnet")

library(verification)

library(pROC) # ROC

library(rms) # nomogram

library(glmnet) # cv.glmnet

setwd("C:/Users/212481425/Desktop/0513_zoumenghong")

df_1<- read.csv("for_stratify_field_strength_1.5.csv",header = T)

df_2<- read.csv("for_stratify_field_strength_3.0.csv",header = T)

df_1<- read.csv("for_stratify_field_strength_1.5.csv",header = T)

df_2<- read.csv("for_stratify_field_strength_3.0.csv",header = T)

df_g<- read.csv("for_stratify_vendor_G.csv",header = T)

df_p<- read.csv("for_stratify_vendor_P.csv",header = T)

df_s<- read.csv("for_stratify_vendor_S.csv",header = T)

roc_1_1<-roc(df_1$label, df_1$CET1_Score,ci=T)

roc_1_2<-roc(df_1$label, df_1$T2_Score,ci=T)

roc_1_3<-roc(df_1$label, df_1$Combine_Score,ci=T)

roc_2_1<-roc(df_2$label, df_2$CET1_Score,ci=T)

roc_2_2<-roc(df_2$label, df_2$T2_Score,ci=T)

roc_2_3<-roc(df_2$label, df_2$Combine_Score,ci=T)

roc_g_1<-roc(df_g$label, df_g$CET1_Score,ci=T)

roc_g_2<-roc(df_g$label, df_g$T2_Score,ci=T)

roc_g_3<-roc(df_g$label, df_g$Combine_Score,ci=T)

roc_p_1<-roc(df_p$label, df_p$CET1_Score,ci=T)

roc_p_2<-roc(df_p$label, df_p$T2_Score,ci=T)

roc_p_3<-roc(df_p$label, df_p$Combine_Score,ci=T)

roc_s_1<-roc(df_s$label, df_s$CET1_Score,ci=T)

roc_s_2<-roc(df_s$label, df_s$T2_Score,ci=T)

roc_s_3<-roc(df_s$label, df_s$Combine_Score,ci=T)

plot(roc_1_1, col=1, main="The ROC curves")

plot(roc_2_1, col=4,add=T)

plot(roc_1_2, col=1, main="The ROC curves")

plot(roc_2_2, col=4,add=T)

plot(roc_3_1, col=1, main="The ROC curves")

plot(roc_3_1, col=4,add=T)

plot(roc_1_3, col=1, main="The ROC curves")

plot(roc_2_3, col=4,add=T)

roc_2_3

roc_1_3

roc_2_2

roc_1_2

roc_1_1

roc_2_1

df_1$label

roc_1_1

roc_1_3<-roc(df_1$label, df_1$Combine_Score,ci=T)

roc_1_3

roc_2_3

df_2$label

roc_g_1

roc_g_3

roc_p_3

roc_s_3

roc_s_2

roc_p_2

roc_p_1

roc_s_1

roc_p_1

roc_p_2

roc_s_2

roc_s_3

roc_p_3

roc_p_1

roc_s_1

roc_g_1

df_g$label

df_g$CET1_Score

roc_g_3

roc_p_1

roc_p_3

df_p$label

df_1$label

df_g$label

roc_1_1

roc_2_1

roc_1_2

roc_2_2

roc_1_3

roc_2_3

roc_1_1<-roc(df_1$label, df_1$CET1_Score,ci=T,print.auc=T)

plot(roc_1_1, col=1, main="The ROC curves",,print.auc=T)

plot(roc_1_1, col=1, main="The ROC curves",print.auc=T)

plot(roc_2_1, col=4,add=T,print.auc=T)

plot(roc_1_2, col=1, main="The ROC curves")

plot(roc_2_2, col=4,add=T)

plot(roc_1_3, col=1, main="The ROC curves")

plot(roc_2_3, col=4,add=T)

roc_1_1

roc_2_1

legend(0.75,0.2,

c("1.5T: AUC=0.500,95% CI=0.226-0.774",

"3.0T: AUC=0.610,95% CI=0.498-0.722"

),

border=3,

cex=1.1,

text.width = 0.65,

col=c(1,4),

lty= 1,

lwd= 3)

plot(roc_1_1, col=1, main="The ROC curves of CET1 Score")

plot(roc_2_1, col=4,add=T)

legend(0.75,0.2,

c("1.5T: AUC=0.500,95% CI=0.226-0.774",

"3.0T: AUC=0.610,95% CI=0.498-0.722"

),

border=3,

cex=1.1,

text.width = 0.65,

col=c(1,4),

lty= 1,

lwd= 3)

roc_1_2

roc_2_2

plot(roc_1_2, col=1, main="The ROC curves of T2 Score")

plot(roc_2_2, col=4,add=T)

legend(0.75,0.2,

c("1.5T: AUC=0.763,95% CI=0.522-1.000",

"3.0T: AUC=0.834,95% CI=0.749-0.919"

),

border=3,

cex=1.1,

text.width = 0.65,

col=c(1,4),

lty= 1,

lwd= 3)

roc_1_3

roc_2_3

plot(roc_1_3, col=1, main="The ROC curves of Combine Score")

plot(roc_2_3, col=4,add=T)

legend(0.75,0.2,

c("1.5T: AUC=0.581,95% CI=0.289-0.873",

"3.0T: AUC=0.563,95% CI=0.433-0.693"

),

border=3,

cex=1.1,

text.width = 0.65,

col=c(1,4),

lty= 1,

lwd= 3)

tiff(file = "C:/Users/212481425/Desktop/ROC_1_600.tiff", res = 1200,width =9000, height = 9000)

plot(roc_1_1, col=1, main="The ROC curves of CET1 Score")

plot(roc_2_1, col=4,add=T)

legend(0.75,0.2,

c("1.5T: AUC=0.500,95% CI=0.226-0.774",

"3.0T: AUC=0.610,95% CI=0.498-0.722"

),

border=3,

cex=1.1,

text.width = 0.65,

col=c(1,4),

lty= 1,

lwd= 3)

dev.off()

tiff(file = "C:/Users/212481425/Desktop/ROC_2_600.tiff", res = 1200,width =9000, height = 9000)

plot(roc_1_2, col=1, main="The ROC curves of T2 Score")

plot(roc_2_2, col=4,add=T)

legend(0.75,0.2,

c("1.5T: AUC=0.763,95% CI=0.522-1.000",

"3.0T: AUC=0.834,95% CI=0.749-0.919"

),

border=3,

cex=1.1,

text.width = 0.65,

col=c(1,4),

lty= 1,

lwd= 3)

dev.off()

tiff(file = "C:/Users/212481425/Desktop/ROC_3_600.tiff", res = 1200,width =9000, height = 9000)

plot(roc_1_3, col=1, main="The ROC curves of Combine Score")

plot(roc_2_3, col=4,add=T)

legend(0.75,0.2,

c("1.5T: AUC=0.581,95% CI=0.289-0.873",

"3.0T: AUC=0.563,95% CI=0.433-0.693"

),

border=3,

cex=1.1,

text.width = 0.65,

col=c(1,4),

lty= 1,

lwd= 3)

dev.off()

plot(roc_g_1, col=1, main="The ROC curves of CET1 Score")

plot(roc_p_1, col=4,add=T)

plot(roc_s_1, col=3,add=T)

roc_g_1

roc_p_1

roc_s_1

plot(roc_g_1, col=1, main="The ROC curves of CET1 Score")

plot(roc_p_1, col=4,add=T)

plot(roc_s_1, col=3,add=T)

legend(0.75,0.2,

c("GE: AUC=0.500,95% CI=0.000-1.000",

"Philips: AUC=0.541,95% CI=0.361-0.721",

"Siemens: AUC=0.608,95% CI=0.456-0.730"

),

border=3,

cex=1.1,

text.width = 0.65,

col=c(1,4,3),

lty= 1,

lwd= 3)

legend(0.75,0.2,

c("GE: AUC=0.500,95% CI=0.000-1.000",

"Philips: AUC=0.541,95% CI=0.361-0.721",

"Siemens: AUC=0.608,95% CI=0.456-0.730"

),

border=3,

cex=1.1,

text.width = 0.65,

col=c(1,4,3),

lty= 1,

lwd= 3)

tiff(file = "C:/Users/212481425/Desktop/ROC_v1_600.tiff", res = 1200,width =9000, height = 9000)

plot(roc_g_1, col=1, main="The ROC curves of CET1 Score")

plot(roc_p_1, col=4,add=T)

plot(roc_s_1, col=3,add=T)

legend(0.75,0.2,

c("GE: AUC=0.500,95% CI=0.000-1.000",

"Philips: AUC=0.541,95% CI=0.361-0.721",

"Siemens: AUC=0.608,95% CI=0.456-0.730"

),

border=3,

cex=1.1,

text.width = 0.65,

col=c(1,4,3),

lty= 1,

lwd= 3)

dev.off()

tiff(file = "C:/Users/212481425/Desktop/ROC_v1_600.tiff", res = 1200,width =9000, height = 9000)

plot(roc_g_1, col=1, main="The ROC curves of CET1 Score")

plot(roc_p_1, col=4,add=T)

plot(roc_s_1, col=3,add=T)

legend(0.75,0.2,

c("GE: AUC=0.500,95% CI=0.000-1.000",

"Philips: AUC=0.541,95% CI=0.361-0.721",

"Siemens: AUC=0.608,95% CI=0.456-0.730"

),

border=3,

cex=1.1,

text.width = 0.65,

col=c(1,4,3),

lty= 1,

lwd= 3)

dev.off()

roc_g_2

plot(roc_g_2, col=1, main="The ROC curves of T2 Score")

plot(roc_p_2, col=4,add=T)

plot(roc_s_2, col=4,add=T)

#---------------------------------- ROC -----------------------------------------

rm(list=ls()) # æ¸ç©º

setwd("C:/Users/212481425/Desktop/0513_zoumenghong")

getwd()

df_1<- read.csv("for_stratify_field_strength_1.5.csv",header = T)

df_2<- read.csv("for_stratify_field_strength_3.0.csv",header = T)

df_g<- read.csv("for_stratify_vendor_G.csv",header = T)

df_p<- read.csv("for_stratify_vendor_P.csv",header = T)

df_s<- read.csv("for_stratify_vendor_S.csv",header = T)

roc_1_1<-roc(df_1$label, df_1$CET1_Score,ci=T)

roc_1_2<-roc(df_1$label, df_1$T2_Score,ci=T)

roc_1_3<-roc(df_1$label, df_1$Combine_Score,ci=T)

roc_2_1<-roc(df_2$label, df_2$CET1_Score,ci=T)

roc_2_2<-roc(df_2$label, df_2$T2_Score,ci=T)

roc_2_3<-roc(df_2$label, df_2$Combine_Score,ci=T)

roc_g_1<-roc(df_g$label, df_g$CET1_Score,ci=T)

roc_g_2<-roc(df_g$label, df_g$T2_Score,ci=T)

roc_g_3<-roc(df_g$label, df_g$Combine_Score,ci=T)

roc_p_1<-roc(df_p$label, df_p$CET1_Score,ci=T)

roc_p_2<-roc(df_p$label, df_p$T2_Score,ci=T)

roc_p_3<-roc(df_p$label, df_p$Combine_Score,ci=T)

roc_s_1<-roc(df_s$label, df_s$CET1_Score,ci=T)

roc_s_2<-roc(df_s$label, df_s$T2_Score,ci=T)

roc_s_3<-roc(df_s$label, df_s$Combine_Score,ci=T)

plot(roc_g_2, col=1, main="The ROC curves of T2 Score")

plot(roc_p_2, col=4,add=T)

plot(roc_s_2, col=3,add=T)

#---------------------------------- ROC -----------------------------------------

rm(list=ls()) # æ¸ç©º

setwd("C:/Users/212481425/Desktop/0513_zoumenghong")

getwd()

df_1<- read.csv("for_stratify_field_strength_1.5.csv",header = T)

df_2<- read.csv("for_stratify_field_strength_3.0.csv",header = T)

df_g<- read.csv("for_stratify_vendor_G.csv",header = T)

df_p<- read.csv("for_stratify_vendor_P.csv",header = T)

df_s<- read.csv("for_stratify_vendor_S.csv",header = T)

roc_1_1<-roc(df_1$label, df_1$CET1_Score,ci=T)

roc_1_2<-roc(df_1$label, df_1$T2_Score,ci=T)

roc_1_3<-roc(df_1$label, df_1$Combine_Score,ci=T)

roc_2_1<-roc(df_2$label, df_2$CET1_Score,ci=T)

roc_2_2<-roc(df_2$label, df_2$T2_Score,ci=T)

roc_2_3<-roc(df_2$label, df_2$Combine_Score,ci=T)

roc_g_1<-roc(df_g$label, df_g$CET1_Score,ci=T)

roc_g_2<-roc(df_g$label, df_g$T2_Score,ci=T)

roc_g_3<-roc(df_g$label, df_g$Combine_Score,ci=T)

roc_p_1<-roc(df_p$label, df_p$CET1_Score,ci=T)

roc_p_2<-roc(df_p$label, df_p$T2_Score,ci=T)

roc_p_3<-roc(df_p$label, df_p$Combine_Score,ci=T)

roc_s_1<-roc(df_s$label, df_s$CET1_Score,ci=T)

roc_s_2<-roc(df_s$label, df_s$T2_Score,ci=T)

roc_s_3<-roc(df_s$label, df_s$Combine_Score,ci=T)

tiff(file = "C:/Users/212481425/Desktop/ROC_1_600.tiff", res = 1200,width =9000, height = 9000)

plot(roc_1_1, col=1, main="The ROC curves of CET1 Score")

plot(roc_2_1, col=4,add=T)

legend(0.75,0.2,

c("1.5T: AUC=0.500,95% CI=0.226-0.774",

"3.0T: AUC=0.610,95% CI=0.498-0.722"

),

border=3,

cex=1.1,

text.width = 0.65,

col=c(1,4),

lty= 1,

lwd= 3)

dev.off()

plot(roc_g_2, col=1, main="The ROC curves of T2 Score")

plot(roc_p_2, col=4,add=T)

plot(roc_s_2, col=3,add=T)

df_g_renew<- read.csv("for_stratify_vendor_G_renew.csv",header = T)

roc_g_2_renew<-roc(df_g_renew$label,df_g_renew$T2_Score,ci=T)

roc_g_2_renew

df_g_renew$T2_Score

df_g_renew$label

roc_g_2_renew<-roc(df_g_renew$label,-1*df_g_renew$T2_Score,ci=T)

roc_g_2_renew

-1*df_g_renew$T2_Score

roc_g_2_renew<-roc(df_g_renew$label,-1*df_g_renew$T2_Score)

roc_g_2_renew

plot(roc_g_2, col=1, main="The ROC curves of T2 Score")

plot(roc_p_2, col=4,add=T)

plot(roc_s_2, col=3,add=T)

roc_g_2

roc_p_2

plot(roc_g_2_renew)

plot(roc_p_2, col=4,add=T)

roc_p_2

roc_s_2

plot(roc_g_2, col=1, main="The ROC curves of T2 Score")

plot(roc_p_2, col=4,add=T)

plot(roc_s_2, col=3,add=T)

legend(0.75,0.2,

c("GE: AUC=0.500,95% CI=0.000-1.000",

"Philips: AUC=0.841,95% CI=0.703-0.978",

"Siemens: AUC=0.832,95% CI=0.741-0.922"

),

border=3,

cex=1.1,

text.width = 0.65,

col=c(1,4,3),

lty= 1,

lwd= 3)

tiff(file = "C:/Users/212481425/Desktop/ROC_v2_600.tiff", res = 1200,width =9000, height = 9000)

plot(roc_g_2, col=1, main="The ROC curves of T2 Score")

plot(roc_p_2, col=4,add=T)

plot(roc_s_2, col=3,add=T)

legend(0.75,0.2,

c("GE: AUC=0.500,95% CI=0.000-1.000",

"Philips: AUC=0.841,95% CI=0.703-0.978",

"Siemens: AUC=0.832,95% CI=0.741-0.922"

),

border=3,

cex=1.1,

text.width = 0.65,

col=c(1,4,3),

lty= 1,

lwd= 3)

dev.off()

plot(roc_g_3, col=1, main="The ROC curves of Combine Score")

plot(roc_p_3, col=4,add=T)

plot(roc_s_3, col=3,add=T)

roc_g_3

roc_p_3

roc_s_3

tiff(file = "C:/Users/212481425/Desktop/ROC_v3_600.tiff", res = 1200,width =9000, height = 9000)

plot(roc_g_3, col=1, main="The ROC curves of Combine Score")

plot(roc_p_3, col=4,add=T)

plot(roc_s_3, col=3,add=T)

legend(0.75,0.2,

c("GE: AUC=0.833,95% CI=0.371-1.000",

"Philips: AUC=0.527,95% CI=0.335-0.719",

"Siemens: AUC=0.532,95% CI=0.375-0.688"

),

border=3,

cex=1.1,

text.width = 0.65,

col=c(1,4,3),

lty= 1,

lwd= 3)

dev.off()

#--------------------------------------------------------------------------------------------

roc.test(roc_1_1,roc_2_1)

roc.test(roc_1_1,roc_2_1)

roc.test(roc_1_2,roc_2_2)

roc.test(roc_1_3,roc_2_3)

roc.test(roc_g_1,roc_p_1)

roc.test(roc_p_1,roc_s_1)

roc.test(roc_g_1,roc_s_1)

roc.test(roc_g_2,roc_p_2)

roc.test(roc_p_2,roc_s_2)

roc.test(roc_g_2,roc_s_2)

roc.test(roc_g_3,roc_p_3)

roc.test(roc_p_3,roc_s_3)

roc.test(roc_g_3,roc_s_3)
